# Electrochromic polyoxometalates for sensing abiotic stress in plants

**DOI:** 10.3389/fpls.2025.1672784

**Published:** 2026-01-02

**Authors:** Ana González, Felipe Pérez-Gordillo, Pablo Alarcón-Guijo, María C. Romero-Puertas, Luisa M. Sandalio, Jose M. Dominguez-Vera

**Affiliations:** 1 Departamento de Química Inorgánica, Instituto de Biotecnología, Facultad de Ciencias, Universidad de Granada, Granada, Spain; 2 Stress, Development and Signaling in Plants, Estación Experimental del Zaidín Consejo Superior de Investigaciones (CSIC), Granada, Spain

**Keywords:** abiotic stress, redox sensor, ascorbic acid, glutathione, polyoxometalates, *Arabidopsis thaliana*, eustress, distress

## Abstract

**Introduction:**

Understanding plant responses to abiotic stress requires an insight into plant redox activity. This study proposes a novel and cost-effective method for assessing the redox state of plants.

**Methods:**

The method utilizes the electrochromic properties of polyoxometalate phosphomolybdic acid hydrate (PMA). PMA is reduced proportionally by glutathione (GSH) and ascorbic acid (AsA), which results in a measurable color change. The validity of this method was confirmed through empirical experimentation in *Arabidopsis thaliana* under conditions of salinity and UV radiation.

**Results:**

Salinity treatments revealed a non-significant, two-phase trend in redox activity with an increase at moderate levels followed by a decrease. UVC radiation led to a substantial decrease in redox activity, indicating distress. In contrast, UVA promoted resilience, also known as eustress. Notably, UVB significantly increased redox activity, suggesting the activation of an emergency antioxidant response.

**Discussion:**

A demonstrable correlation has been identified between the redox activity of plants and various stress types. This correlation facilitates the classification of responses into two distinct categories: adaptive eustress and detrimental distress. This advancement contributes to the enhancement of plant metabolic and stress tolerance evaluation.

## 1 Introduction

Plants, like all other aerobic organisms, need oxygen to live. However, oxygen can be also toxic to them due to reactive oxygen species (ROS) (Halliwell and Gutteridge, 2015). ROS in excess are considered as harmful species associated with oxidative damage in cells, a phenomenon referred to as oxidative stress (Dumanović et al., 2021; Mishra et al., 2023). The oxidative stress should not be defined exclusively as an increase in ROS. Instead, it should be defined as an anomaly between the redox signal of plants caused by an imbalance between oxidant species and antioxidant defenses with an inclination toward oxidants (Jones, 2006).

The overproduction of ROS is frequently linked to abiotic stress, defined as environmental conditions such as drought, UV radiation, salinity, extreme temperatures, and heavy metals, which induce a decline in plant growth and elicit physiological and biochemical alterations (Talbi et al., 2015; Hafsi et al., 2022; Mishra et al., 2023). The distinction between physiological oxidative stress and extreme oxidative stress hinges on the intensity of the stressor. Physiological oxidative stress, characterized by low exposure to oxidative stress, eustress, enables the plant to manage certain aspects of redox signaling and even generate acclimatization to the stress. In contrast, extreme oxidative stress, marked by high exposure to oxidative stress, or distress, leads to the disruption of the redox machinery, resulting in severe damage to specific biomolecules (Sies, 2018; Cejudo et al., 2021).

In response to these ROS, plants have developed an antioxidant defense system (AOS). This includes a variety of antioxidant molecules. Depending on their chemical nature, antioxidants can be classified as enzymatic or non-enzymatic (Dumanović et al., 2021; Mishra et al., 2023). Superoxide dismutase (SOD), ascorbate peroxidase (APX), catalase (CAT), glutathione peroxidase (GPX), monodehydroascorbate reductase (MDHAR), dehydrogenase (DHAR), glutathione reductase (GR), and glutathione S-transferase (GST) are the major antioxidant enzymes in plants (Mittler et al., 2011; Halliwell and Gutteridge, 2015). Conversely, the group of non-enzymatic antioxidants encompasses a series of low molecular weight water-soluble compounds, predominantly ascorbic acid (AsA) and glutathione (GSH), along with liposoluble compounds such as tocopherols, tocotrienols, and β-carotenoids (Halliwell and Gutteridge, 2015). Furthermore, other biomolecules, including amino acids, sugars, pigments, flavonoids, and terpenoids, have been shown to possess antioxidant activity (Halliwell and Gutteridge, 2015; Dumanović et al., 2021).

Among the antioxidants present in plants, AsA and GSH stand out as the most abundant (Foyer and Noctor, 2011). Concentrations of AsA and GSH exhibit variation among species, plant tissues and organelles (Noctor and Foyer, 2016). However, the AsA content typically exhibits a tenfold increase over the GSH content. For instance, a typical leaf content of 3 mmol·g^-1^ and 0.3 mmol·g^-1^ fresh weight in AsA and GSH, respectively, has been estimated for *Arabidopsis* under standard and unstressed conditions (Noctor and Foyer, 2016).

The presence of AsA and GSH is essential for the scavenging of electrons, which results in the removal of ROS. It has been established that both compounds collaborate with APX, MDHAR, DHAR and GR in the metabolization of hydrogen peroxide (H_2_O_2_). This metabolic process is referred to as the AsA-GSH cycle (Halliwell and Gutteridge, 2015). In this cycle, hydrogen peroxide is converted to water through an enzymatic process in which APX oxidizes two molecules of AsA to dehydroascorbate (DHA), which in turn uses GSH and DHAR to recover AsA. Consequently, glutathione disulfide (GSSG), the oxidized form of GSH, is produced; however, it is rapidly recovered to GSH by GR (Halliwell and Gutteridge, 2015; Dumanović et al., 2021).

Different abiotic stress conditions from nutritional deficiency to heavy metals, heat, drought or salinity, between others, are characterized by an increase in oxidants, and these conditions have been demonstrated to disrupt the concentrations of AsA, GSH, APX, DHAR, and GR (Talbi et al., 2015; Gupta et al., 2017; Peco et al., 2020; Collado-Arenal et al., 2024). This disruption is typically associated with an increase in the expression of enzymes associated to the AsA-GSH cycle, although it would be dependent on the type of stress, experimental conditions and plant species (Gupta et al., 2013, 2017; Pandey et al., 2017; Hasanuzzaman et al., 2020; Peco et al., 2020).

The content of AsA and GSH has been considered as excellent markers of the redox state of the plants under stress conditions and they have been used as parameters of oxidative stress and tolerance to environmental adverse conditions in plants and animals. The determination of AsA and GSH in plants is conducted individually. Each molecule requires a different procedure, with multiple options available in each instance. In the case of GSH, the determination can be performed under chromatography, e.g., HPLC with fluorescence detection (Vojtovič and Petřivalský, 2024). Alternatively, a conventional spectrophotometric approach is frequently employed, which is based on the oxidation of GSH by 5,5′-dithio-bis(2-nitrobenzoic acid) (DTNB) to a yellow derivative known as 5′-thio-2-nitrobenzoic acid (TNB). This method also affords the GSSG content by initially reducing this molecule to GSH by GR in the presence of NADPH and subsequently permitting the GSH to react once more with DTNB. The absorption band of TNB at 412 nm has been demonstrated to be directly related to the concentration of GSH (Sahoo et al., 2017). Conversely, the quantification of AsA is frequently conducted through spectrophotometric and chromatographic methods, with some instances of titration and amperometric techniques also employed (Raghu et al., 2007).

The development of a methodology capable of detecting the total redox activity of the plant without the necessity of applying specific treatments to each individual species involved in this process would be of significant interest. The implementation of such a technique would facilitate the monitoring of the impact of diverse stressors on the plant. Electrochromic sensors based on polyoxometalates could be a useful tool for addressing this challenge. Polyoxometalates are molecular fragments of transition metal oxides such as Mo, W, V, Nb and Ta combined with P, Si and Al as heteroatoms. These compounds possess a multitude of properties that render them suitable for utilization in a variety of disciplines, including magnetism, materials science, electrochemistry, catalysis, crystallography, and medicine (Gumerova and Rompel, 2018; Lan et al., 2021; Yenel et al., 2021; Housaindokht et al., 2022; Maity et al., 2022; Yıldırım et al., 2022; Woźniak-Budych et al., 2023; Zhang et al., 2023; Wang et al., 2024; Akbar Hussaini et al., 2025). POMs possess an additional property that merits attention: their capacity to function as electrochromic materials due to their unique redox properties (Coronado et al., 2005; Yenel et al., 2021; Yıldırım et al., 2022; Akbar Hussaini et al., 2025). In this sense, we explored the correlation between the redox activity of probiotic bacteria and their metabolic strength (González et al., 2015, 2020; Sabio et al., 2021) using this kind of electrochromic sensors. In such a scenario, the redox activity could be monitored by the reduction reaction of the polyoxometalate PMO (Na_6_[P_2_Mo_VI18_O_62_]) with reductants excreted by the probiotic bacteria. Upon reduction, PMO changes color from colorless to blue, and the intensity of this blue color directly reflects the extent of the reduction (González et al., 2015, 2020; Sabio et al., 2021). Additionally, as the extinction coefficient of reduced POMs is elevated, quantification of small concentrations of other reducing species was also performed (González et al., 2015; Zou et al., 2018; Sánchez et al., 2021). In this study, we expand this strategy by evaluating the potential reduction of POMs by compounds present in the antioxidant mechanism of plants, such as AsA and GSH. The central hypothesis of this study is that this reduction could serve as a screening method to assess the impact of redox activity on abiotic stresses experienced by plants. This approach is expected to facilitate the identification of eustress situations or even transitions from eustress to distress under changing conditions. Despite the recent development of powerful nanosensors for agricultural applications (Giraldo and Kruss, 2023), no sensor has yet been described that can identify these types of scenarios. To the best of our knowledge, this is the first report to apply the electrochromic properties of phosphomolybdic acid (PMA) as a cost-effective screening sensor for monitoring the redox pool in response to salinity and UV light abiotic stresses in *Arabidopsis thaliana*. The chromatic shift functions as a qualifiable metric, thereby offering a means to ascertain the redox level of AsA and GSH in plants.

## 2 Material and methods

### 2.1 Reagents

All reagents used were purchased from Sigma–Aldrich at the highest purity available. PMO (Na_6_[P_2_Mo_VI18_O_62_]), was synthetized as previously reported (Strandberg et al., 1975). Aqueous solutions were prepared with ultrapure water (18.2MΩcm, bacteria < 0.1 CFU mL−1 at 25°C, Milli-Q, Millipore).

### 2.2 POMs reduction by GSH

Three POMs were investigated: phosphomolybdic acid hydrate (PMA, H_3_[P(Mo_3_O_10_)_4_] · xH_2_O), phosphotungstic acid hydrate PTA (H_3_[P_4_(W_12_O_40_) · xH_2_O), and the synthetic PMO. To study their reactions with GSH, aqueous solutions of each POM were prepared at concentrations of 1, 5, and 10 mM. These POM solutions were then mixed with varying volumes of a 10 mM aqueous solution of GSH in 96-well plates. The volumes of GSH solution were carefully adjusted to achieve final GSH concentrations of 0, 0.1, 0.25, 0.5, 0.75, and 1 mM in each well. All reactions were carried out in a final total volume of 200 µL per well. Samples were prepared in triplicates. After 30 minutes at room temperature, the UV-visible absorption spectra of the samples were recorded from 450 to 900 nm using a CLARIOstar plate reader (BMGLabtech, Ortenberg, Germany).

### 2.3 Optimization of PMA reduction by GSH

To optimize the PMA reaction with GSH, aqueous solutions of PMA were prepared at concentrations of 10, 15, and 20 mM. These PMA solutions were then mixed with varying volumes of a 10 mM aqueous solution of GSH in 96-well plates. The volumes of GSH solution were carefully adjusted to achieve final GSH concentrations of 1, 5, 10, 25, 50, 75, 100, 250, 500, 750 and 1000 µM in each well. All reactions were carried out in a final total volume of 200 µL per well. Samples were prepared in triplicates. After 30 minutes at room temperature, the UV-visible absorption spectra of the samples were recorded from 450 to 900 nm using a CLARIOstar plate reader (BMGLabtech, Ortenberg, Germany).

### 2.4 PMA reduction by GSH, glutathione disulfide (GSSG), AsA, dehydroascorbate (DHA), H_2_O_2_, flavin adenine dinucleotide (FAD), caffeic acid (CA) and phenol (PH)

A consistent 80 µL of 25 mM aqueous PMA solution was used for each sample. This PMA solution was then mixed with varying volumes of other compounds in a 96-well plate. Specifically, GSH, GSSG, AsA, DHA, H_2_O_2_, FAD, CA and PH were prepared as 1 mM solutions in 10 mM HCl. These solutions were added in volumes of 0, 1, 2, 5, 10, 15, 20, or 40 µL to the wells containing the PMA. To ensure all wells had a uniform total volume, 20 µL of Milli-Q water was added. Additionally, a calculated volume of 10 mM HCl was included in each well to bring the final volume of each sample up to 200 µL. This careful adjustment of HCl volume accounted for the varying amounts of the 1 mM compound solutions (GSH, GSSG, etc.) that were also prepared in 10 mM HCl. This method resulted in final concentrations of the added compounds (GSH, GSSG, AsA, DHA, H_2_O_2_, FAD, CA and PH) ranging from 0, 5, 10, 25, 50, 75, 100, to 200 µM. The final PMA concentration in all samples was 10 mM. Samples were prepared in triplicates. After 30 minutes at room temperature, the UV-visible absorption spectra of the samples were recorded from 450 to 900 nm using a CLARIOstar plate reader (BMGLabtech, Ortenberg, Germany).

### 2.5 Plant material and growth conditions for salinity treatment

*Arabidopsis thaliana* ecotype Columbia‐0 (Col‐0) seeds (WT) were surface sterilized and stratified for 48 h at 4°C and then sown on Hoagland (Hoagland and Arnon, 1938) nutrient 0.5 x solid medium containing 3% sucrose (w/v) and 0.8% phytoagar (w/v). The plants were then grown vertically at 22°C under 100 µE irradiance, 60-65% relative humidity in 16 h of light and 8 h of darkness for 13 days. After growing, *A. thaliana* plants were transferred to Petri dishes with 0.5 x liquid Hoagland medium and NaCl at different concentrations (50, 100, 150 and 200 mM) and then grown for 5 days. A non-NaCl containing medium was used as control (NaCl 0 mM). The time just before salt addition was set as the 0 h experimental point.

### 2.6 Plant material and growth conditions for UV treatment

WT (Col‐0) *Arabidopsis thaliana* seeds were surface sterilized and stratified for 48 h at 4°C and then sown on Hoagland (Hoagland and Arnon, 1938) nutrient 0.5 x solid medium containing 3% sucrose (w/v) and 0.8% phytoagar (w/v). The plants were then grown as described before, for 18 days. The plates were then exposed to UVA, UVB, UVC or UVA+UVB irradiation at different times (10, 30 and 120 min) at a distance of 113 mm in a UV CABIN 3C-620 PT (BCB, S.L). The cabin utilized three low-pressure fluorescent lamps of each type (UVA, UVB, UVC) with reflectors for maximum homogenization. The spectral ranges were narrower compared to those of the solar irradiation, with *λ*max at 365 for UVA, 311 nm for UVB, and 254 nm for UVC. The technical specifications for the cabin indicate an average irradiance of 12 W·m^-2^ which corresponds to doses of 7.2, 21.6, and 86.4 KJ·m^-2^ at the times used (10, 30, and 120 min). The combination of UVA+UVB is the sum of the individual contributions of each type of radiation. A non-irradiated plate was taken as control (0 min UV). At each sampling time and UV treatment, plants were weighted and collected in groups of around 100 mg, immediately frozen in liquid nitrogen and conserved at −80°C.

### 2.7 Analysis of redox activity in plants after abiotic stress

After 5 days of salinity treatment, plant samples were weighed and collected in groups of around 100 mg. Ice-cooled HCl 10 mM was added to each extract (1/3, m/v) and samples were homogenized with a pistil. The homogenate was centrifuged at 19,000 g for 30 min at 4°C, and the supernatant was collected for analysis. A total of 6 extracts were collected for the 0 and 100 mM NaCl treatment, 5 extracts were collected from the 50 and 150 mM NaCl treatment and 4 extracts were collected from the 200 mM NaCl treatment.

In case of UV treatment, -80°C samples were ground in a cold mortar with liquid nitrogen and homogenized with ice-cooled HCl 10 mM (1/3, m/v). The homogenate was centrifuged at 19,000 g for 30 min at 4°C, and the supernatant was collected for analysis. A detailed description of the number of extracts (n) obtained at each condition is shown in **Table 1**.

**Table 1:** Number of acid extracts of *A. thaliana* obtained after UV treatment.

UV type	Time (min)	Number of extracts (n)
Control (no UV)	0	7
UVA	10	3
	30	6
	120	6
UVB	10	6
	30	7
	120	4
UVC	10	5
	30	5
	120	2
UVA+UVB	10	7
	30	6
	120	2

Then, the redox activity of the extract was analyzed in triplicates. A consistent 80 µL of 25 mM aqueous PMA solution was used for each sample. This PMA solution was then mixed with 100 µL of each different plant extract in a 96-well plate. To ensure all wells had a uniform total volume of 200 µL, 20 µL of Milli-Q water was added. The final PMA concentration in all samples was 10 mM. After 30 minutes at room temperature, the UV-visible absorption spectra of the samples were recorded from 450 to 900 nm using a CLARIOstar plate reader (BMGLabtech, Ortenberg, Germany). The unstressed control sample (0 mM NaCl or 0 min UV) was used as the reference condition for all treatments. It has to be noted that the inherent absorbance of the crude extract is considered to be independent of the signal being monitored, as the PMA assay is highly selective for the formation of the PMA^red^ product at 852 nm. This methodological approach ensures that the reported changes in absorbance strictly reflect the dynamic, stress-induced shift in the redox capacity, as opposed to static differences in background absorbance.

### 2.8 Data treatment and statistical analysis

Each biological extract was analyzed in technical triplicate with PMA. For each set of extract-PMA triplicates, the Dixon’s Q test at 95% and 99% was performed to eliminate possible outliers. Subsequently, with the valid data, the mean and standard deviation were calculated. Then, to establish significant differences between the different saline treatments or exposure times in each UV radiation type, a one-way ANOVA analysis was carried out. For those experimental groups where significant differences were found, a two-sample t-test assuming unequal variances and the Bonferoni’s correction was applied. For the evaluation of redox activity under conditions of salinity, ANOVA analysis was supplemented with the Games-Howell *post-hoc* test. This method was selected to perform robust pairwise comparisons, appropriate for groups with potentially unequal sample sizes and variances, with a significance level of p = 0.05.

## 3 Results

### 3.1 Reactions of POMs with the different antioxidants and fine-tuning of the method

#### 3.1.1 Evaluation of POM reduction by GSH

In the initial approach, we sought to ascertain the potential reaction of GSH with POMs. To this end, we mixed three distinct POMs at varying concentrations and times with increasing concentrations of GSH ranging from 0.1 to 1 mM. These POMs include the synthetic PMO (Na_6_[P_2_Mo_VI18_O_62_]), and the commercial PMA (H_3_[P_4_Mo_12_O_40_] · xH_2_O) and PTA (H_3_[P_4_(W_12_O_40_) · xH_2_O). The UV-visible spectral profile of each sample was collected at 30 minutes following the addition of the corresponding POM. The results of the study indicated that GSH functions as an effective electron donor for PMO and PMA within the range of 0.1 to 1 mM GSH concentrations. This conclusion was supported by the observation of the typical change of color of PMO and PMA from colorless to intense blue. The corresponding UV-visible absorption bands of PMO^red^ and PMA^red^ were found at 780 and 852 nm, respectively, as detailed in the **Supplementary Material** (**Supplementary Figures S1-S6**). However, PTA reduction and subsequent color observation was not observed at any of the GSH concentrations tested. PTA was then discarded as a potential sensor of GSH.

**Figure 1:**
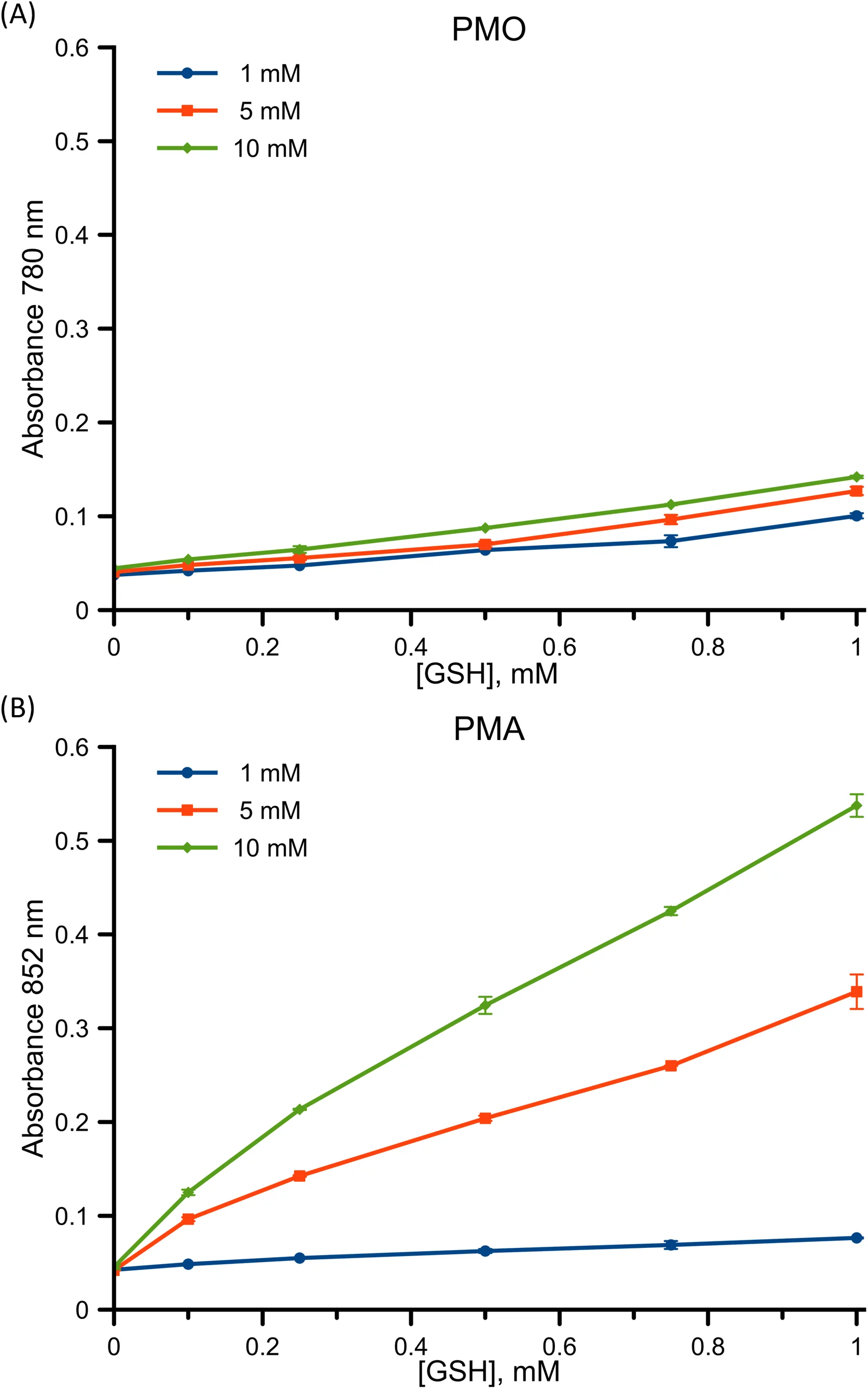
Concentration-dependent reduction of PMO **(A)** and PMA **(B)** by glutathione (GSH). Maximum absorbance at 30 minutes after polyoxometalate (POM) addition to samples containing increasing GSH concentrations (0 to 1 mM) is shown. POM concentrations were 1 mM (blue lines with circles), 5 mM (orange lines with squares), and 10 mM (green lines with diamonds). Error bars represent standard deviations (n= 3 technical replicates per concentration).

The spectral profile analysis for PMO^red^ and PMA^red^ demonstrated an increase in color intensity over time at all concentrations of GSH, with higher concentrations of POM resulting in greater color. Thus, **Figure 1** shows how the maximum absorbance of PMO^red^ and PMA^red^ increases versus GSH concentration. In particular, the absorbance recorded for PMA^red^ is higher than that recorded for PMO^red^. Whereas PMO^red^ absorbance is very similar for all the tested concentrations, for PMA^red^, differences in the signal obtained at different concentrations are more important, being the signals obtained for PMA 10 mM the most effective.

#### 3.1.2 Optimization of PMA concentration and GSH detection range

**Figure 2:**
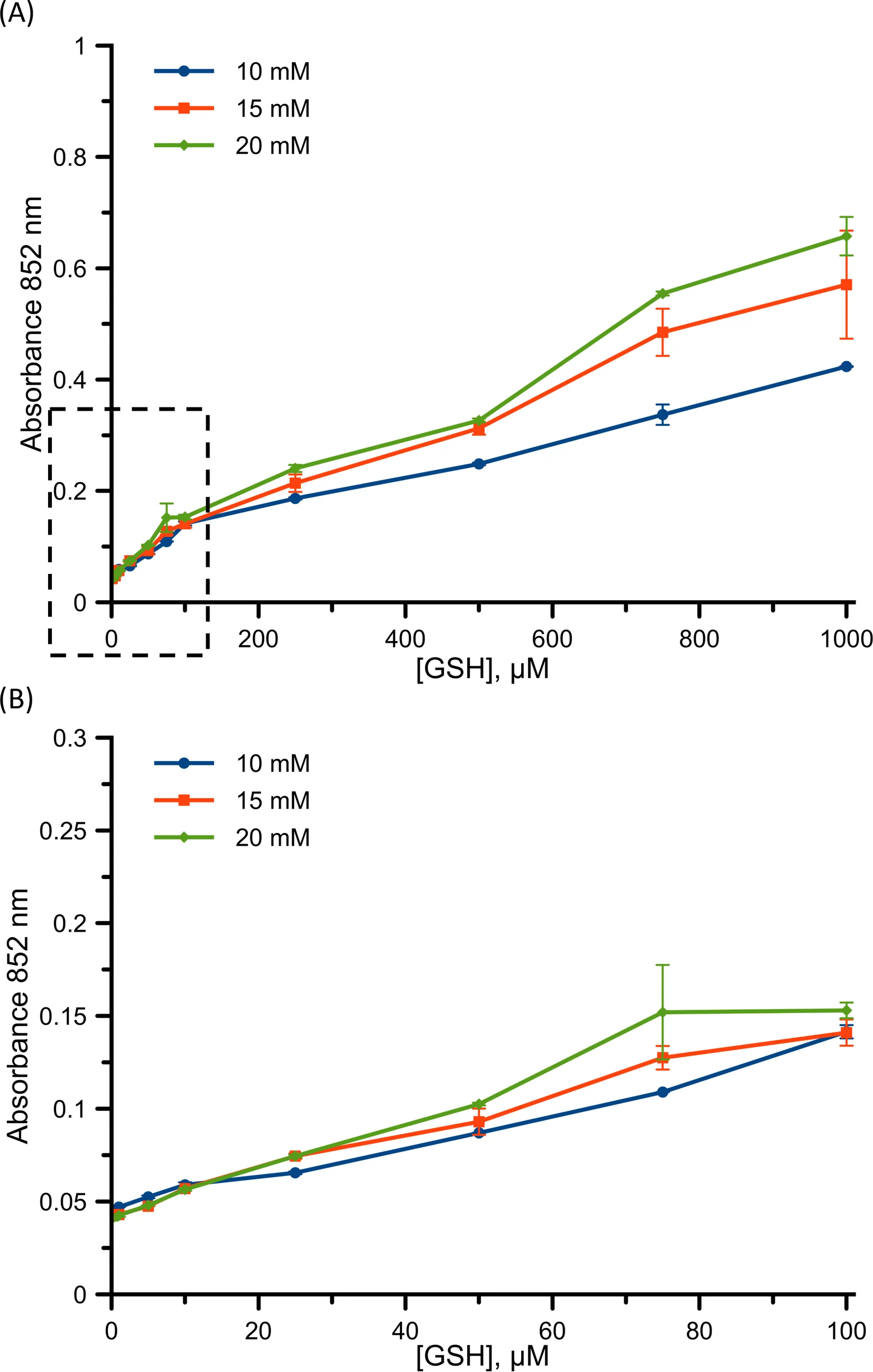
Optimization of phosphomolybdic acid (PMA) concentration for glutathione (GSH) detection. **(A)** Maximum absorbance at 852 nm measured 30 minutes after PMA addition to samples containing GSH concentrations from 0 to 1000 µM. PMA concentrations used were 10 mM (blue lines with circles), 15 mM (orange lines with squares), and 20 mM (green lines with diamonds). **(B)** Enlarged view of the boxed area in **(A)**, highlighting maximum absorbance for GSH concentrations from 0 to 100 µM. Error bars represent standard deviations (n= 3 technical replicates per concentration).

A second experiment was conducted to address lower GSH concentrations, which now ranged from 1 to 1000 µM, in accordance with the hypothesis that higher concentrations of PMA would result in lower concentrations of GSH. To test this hypothesis, PMA concentrations of 15 and 20 mM were tested in the experiment. As anticipated, the comprehensive spectral profile of these novel samples once again exhibited a band centered at 852 nm that was more pronounced at elevated GSH concentrations and extended reaction times between GSH and PMA (see **Supplementary Figure S7**). As illustrated in **Figure 2A**, the maximum values at 852 nm were documented for the entire range of GSH concentrations that were assayed for each PMA concentration. The disparities in PMA concentrations were more pronounced at elevated GSH concentrations, with the maximum absorption recorded at GSH 1 mM and PMA 20 mM. However, for lower GSH concentrations (1 to 100 µM), slight differences were observed between the PMA concentrations of 10, 15, and 20 mM (**Figure 2B**). Furthermore, it is imperative to acknowledge that the level of absorption attained with GSH at a concentration of 1 µM exhibited a marginal increase compared to the control sample devoid of GSH. In accordance with these results, 10 mM PMA concentration was selected as the ideal for determining GSH concentrations of at least 5 µM.

#### 3.1.3 Feasibility of the method in real samples

At this point the feasibility of employing this method for the quantification of GSH in authentic samples was contemplated. However, several factors must be taken into consideration prior to the analysis of actual samples: i) Firstly, it is imperative to ascertain the effect of HCl, a strong acid, on the PMA-GSH reaction, given that the extraction of plant extracts necessitates the utilization of such strong acids. In this line of research, the determination of GSH was conducted within the range of 5 to 200 µM, with HCl 10 mM utilized as the solvent. The findings indicated a favorable outcome, as the maximum absorption at 852 nm exhibited by PMA at varying GSH concentrations was observed to slightly higher than that in the aqueous solution. This outcome also demonstrated enhanced linearity (see **Supplementary Figure S8**). ii) Secondly, other molecules with redox activity may be present in plant extracts in acidic media. Among all the possibilities, as described above, AsA also participates in the defense against ROS in plants and its presence in these acidic extracts is guaranteed. Therefore, it is necessary to evaluate the reducing capacity of AsA against PMA under the conditions established for GSH.

**Figure 3:**
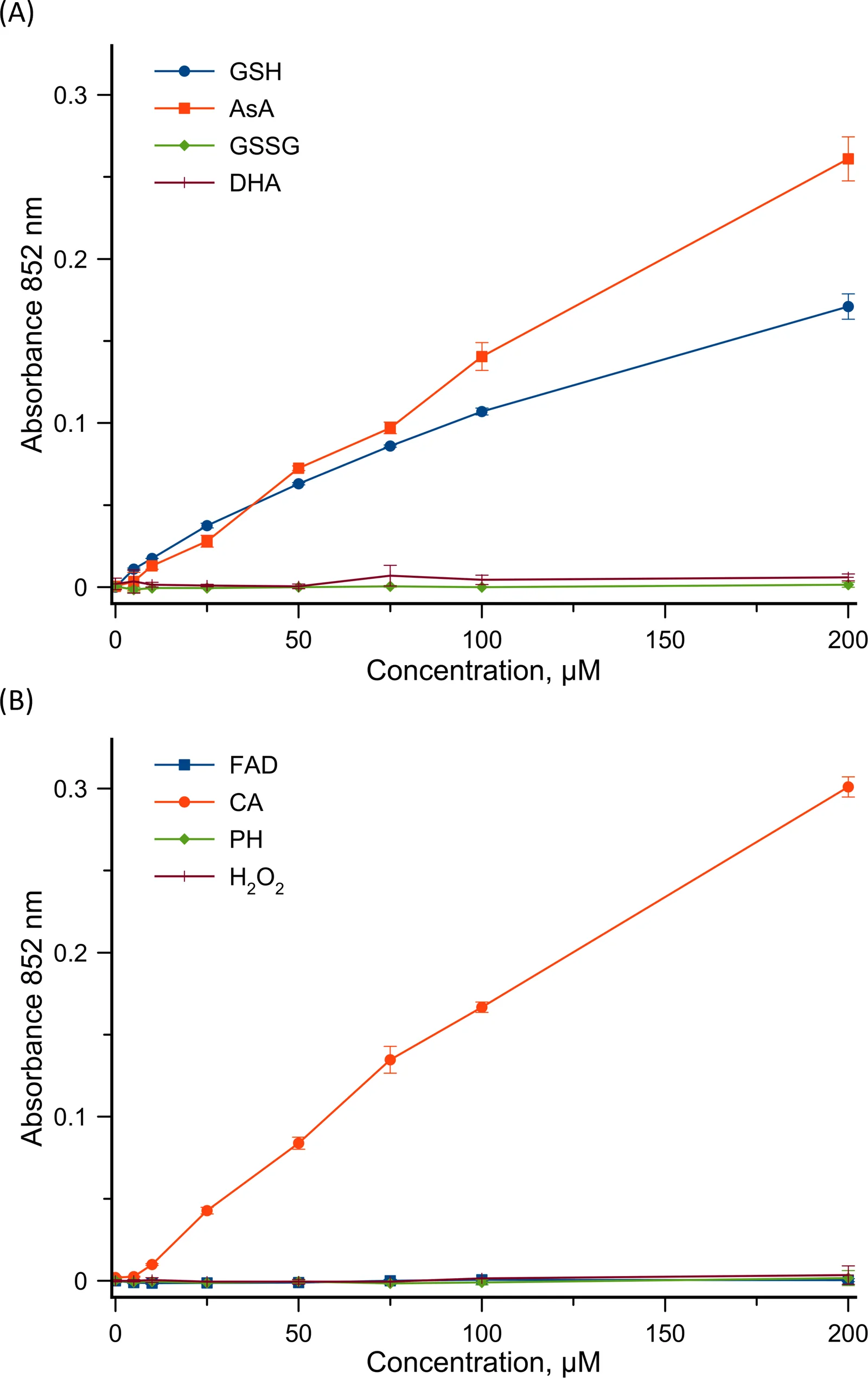
Specificity of phosphomolybdic acid (PMA) reduction by plant antioxidants and lack of reduction by their oxidized forms or hydrogen peroxide. **(A)** Maximum absorbance at 852 nm from samples containing increasing concentrations (0 to 200 µM) of glutathione (GSH, blue line with circles), ascorbic acid (AsA, orange line with squares), glutathione disulfide (GSSG, green line with diamonds), dehydroascorbate (DHA, red-crimson line with crossroads) reacted with PMA 10 mM in 10 mM HCl for 30 minutes. **(B)** Maximum absorbance at 852 nm from samples containing increasing concentrations (0 to 200 µM) of, flavin adenine dinucleotide (FAD, blue line with circles), caffeic acid (CA, orange line with squares), phenol (PH, green line with diamonds) and hydrogen peroxide (H_2_O_2_, red-crimson line with crossroads), reacted with PMA 10 mM in 10 mM HCl for 30 minutes. Error bars represent standard deviations (n= 3 technical replicates per concentration).

**Figure 3A** shows the results obtained after the reaction between PMA 10 mM and GSH and AsA in concentrations from 5 to 200 µM using HCl 10 mM as solvent. The UV-visible absorbance intensity for PMA^red^ at each AsA resulted to be slightly higher than that of GSH, but very similar as both molecules showed the same UV-visible spectral profile with the band centered at 852 nm (see **Supplementary Figure S9**). In the same line, it was deemed imperative to assess the interaction of the oxidation products of both GSH and AsA, that is, dehydroascorbate (DHA) and glutathione disulfide (GSSG), with PMA. **Figure 3A** shows that, as expected, no absorbance was registered after mixture of DHA or GSSG with PMA, clearly indicating no interference reaction to sense GSH and AsA.

In addition to the evaluation of AsA reducing capacity, other minor antioxidant molecules present in *Arabidopsis thaliana* extracts, such as phenolics, catechols, and flavins, were also considered (Fitzpatrick et al., 2007; Roje, 2007; Sandoval et al., 2008; Jaini et al., 2017). To assess their potential contribution to PMA reduction, the same assay was performed with flavin adenine dinucleotide (FAD), caffeic acid (CA), and phenol (PH). These specific molecules were selected because FAD constitutes the major form of flavins, representing approximately 70–90% of the total flavin pool in plants (Fitzpatrick et al., 2007; Roje, 2007; Sandoval et al., 2008). Furthermore, CA has been identified as one of the most prevalent catecholic compounds reported in *A. thaliana* (Jaini et al., 2017). Moreover, the use of PH as a model compound enabled the representation of total phenolic compounds. The results presented in **Figure 3B** demonstrate that CA was the only substance capable of reducing PMA at concentrations ranging from 10 to 200 µM. At the 5 µM concentration, the signal was indistinguishable from the control (0 µM of CA).

Furthermore, as explained above, during oxidative stress processes, when the defense mechanism in a plant, i.e. the AsA-GSH cycle, is activated, H_2_O_2_ is detoxified to H_2_O. Thus, the presence of H_2_O_2_ in a plant acid extract can also be considered, and since H_2_O_2_ is a molecule that also exhibits redox properties, assessing its potential ability to reduce PMA was also fundamental to our study. Since there is no consensus on the amount and concentration of H_2_O_2_ in a plant due to the difficulties associated with its quantification (Noctor and Foyer, 2016; Smirnoff and Arnaud, 2019), we decided to test the same concentration range as for GSH and AsA and found that in no case H_2_O_2_ was able to reduce PMA at a concentration of 10 mM (**Figure 3B**).

### 3.2 Evaluation of the redox activity of *A. thaliana* subjected to abiotic stress conditions using PMA: salinity and UV radiation

In view of the above results, we considered the possibility of developing a method that would allow us to evaluate the redox activity of a plant under certain conditions, whether physiological or abiotic stress. Taking advantage of the standard conditions established for this signal, a correlation can be established between fluctuations in PMA^red^ color and varying levels of GSH and AsA within the plant. These fluctuations are indicative of a change in the redox balance of the plant as a consequence of abiotic stress conditions. Under this hypothesis we selected *Arabidopsis thaliana* seedlings as a model to establish the hypothesized screening method. Specifically, we have evaluated the expected changes in the redox activity of the plants when subjected to two different abiotic stresses: salinity and UV radiation (UV).

#### 3.2.1 Redox activity in response to salinity stress

*A. thaliana* seedlings were growth for 2 weeks in growth chamber and standard Hoagland media. Subsequently, we transferred the plants to Petri dishes containing liquid Hoagland medium with different concentrations of NaCl, using a plate without NaCl and only media as a control. Since previous studies have shown that *A. thaliana* inhibits its growth in the presence of NaCl concentrations from 1 to 100 mM, being the complete inhibition when the concentration of 200 mM is reached (Gill and Tuteja, 2010; Foyer and Noctor, 2011; Xie et al., 2011), the NaCl concentrations selected for our experiment were 50, 100, 150 and 200 mM. In this way, we aimed to cover situations that would provoke salt stress in the plant at different levels to try to monitor the redox activity during the transition from eustress to distress.

**Figure 4:**
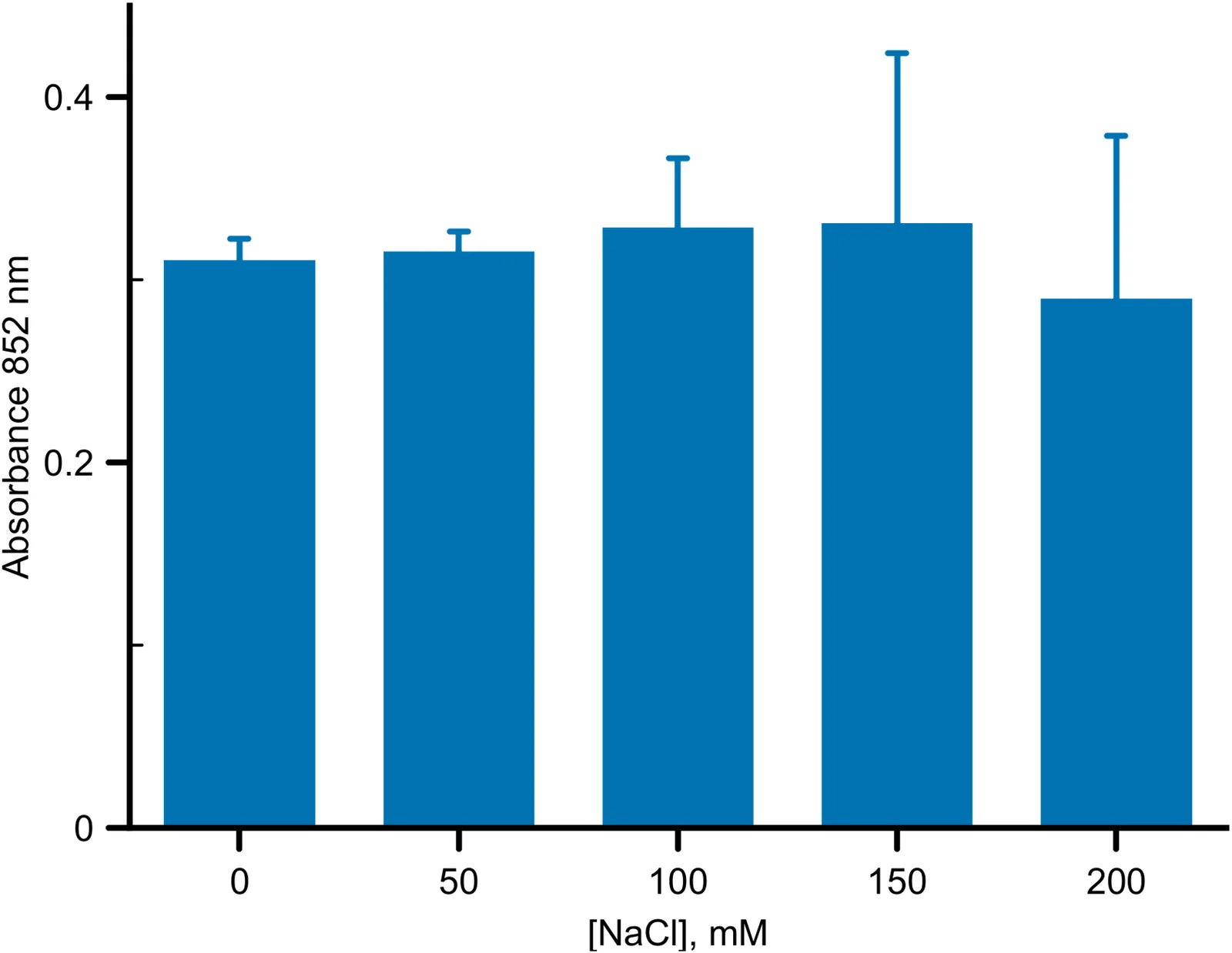
Impact of increasing salinity on the redox activity of *Arabidopsis thaliana* plant extracts. Maximum absorbance at 852 nm, reflecting redox activity, was measured after exposure to various NaCl concentrations (0–200 mM). Six biological replicates (n = 6) were analyzed for 0 and 100 mM NaCl treatments; five replicates (n = 5) for 50 and 150 mM; and four replicates (n = 4) for 200 mM. Each biological sample was measured in technical triplicate. Bars show the mean of these replicates, with error bars indicating standard deviation. ANOVA revealed no statistically significant differences among treatments (F(4, 21) = 1.58, p = 0.215). *Post-hoc* analysis using the Games-Howell test was performed to quantify pairwise comparisons, but confirmed the lack of statistical significance between all treatment groups and the control (p > 0.05). Therefore, no statistical indicators are shown in this figure.

Plants were maintained in the growth chamber with NaCl for 5 days and then subjected to acid extraction with HCl 10 mM. The acidified supernatants obtained were reacted 30 minutes with PMA to measure the redox activity of the extracts. As demonstrated in **Figure 4**, the implementation of our methodology enabled the observation of a trend: an initial increase in redox activity, presumably associated with GSH and AsA, in response to NaCl concentrations reaching up to 150 mM, suggesting an antioxidant response under moderate salinity, with a decrease at 200 mM NaCl, remaining the redox activity at lower levels than those displayed by the control (0 mM NaCl). However, ANOVA revealed no statistically significant differences in the absorbance values recorded across any of the tested conditions (F(4, 21) = 1.58, p = 0.215). To quantify this trend, a Games-Howell *post-hoc* analysis was performed. This analysis confirmed the absence of statistical significance, thereby demonstrating that no salt concentration was significantly different from the control (100 mM vs. Control: p = 0.805; 150 mM vs. Control: p = 0.985; 200 mM vs. Control: p = 0.658). Therefore, although a two-phase pattern is observed, redox activity under salinity of 0–200 mM cannot be considered statistically different in this study. For a detailed overview of the values collected for each extract at each NaCl concentration, refer to the **Supplementary Material** (**Supplementary Figure S10**).

#### 3.2.2 Redox response to UV radiation

Ultraviolet (UV) radiation is known to modulate redox homeostasis in plants by inducing reactive oxygen species (ROS) and triggering antioxidant responses, particularly involving GSH and AsA (Rozema et al., 1997; Gao and Zhang, 2008; Liu et al., 2023). Based on this, we evaluated redox activity in *A. thaliana* exposed to UVA, UVB, UVC, and combined UVA+UVB treatments.

**Figure 5:**
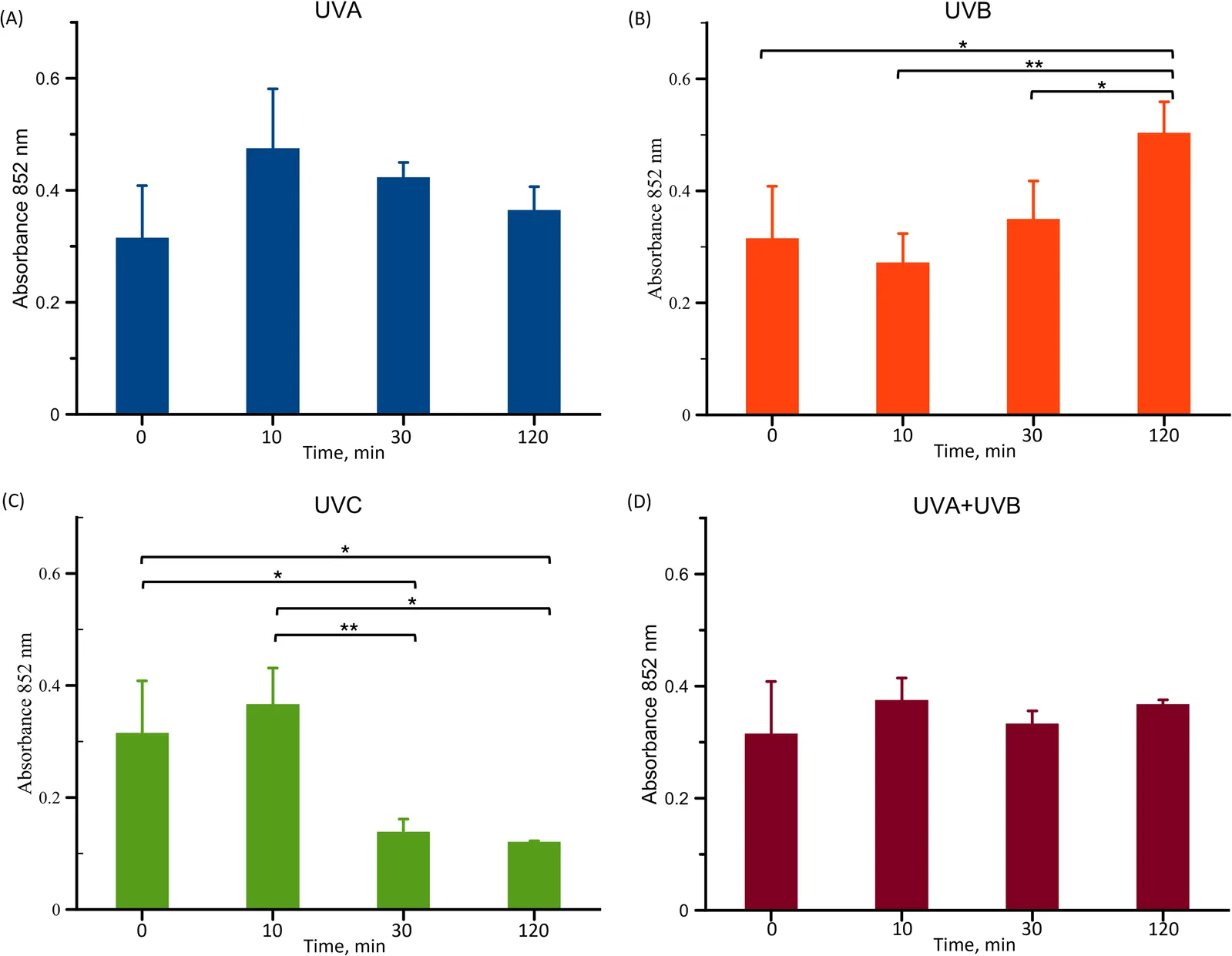
Effect of various UV radiation types and exposure durations on the redox activity of *Arabidopsis thaliana* plant extracts. Maximum absorbance at 852 nm was measured in acidic extracts after exposure to different UV treatments and time points, compared to unexposed control plants (0 min). Panels show results for: **(A)** UVA, **(B)** UVB, **(C)** UVC, and **(D)** UVA + UVB. Each bar represents the mean redox activity from n independent biological replicates (n = 2–7 per condition; see **Table 1** in Materials and Methods). Each biological extract was measured in technical triplicate. Error bars indicate standard deviations. One-way ANOVA revealed significant effects for: UVA: F(3, 18) = 4.726, p = 0.013; UVB: F(3, 20) = 8.991, p = 0.00057; UVC: F(3, 15) = 13.409, p = 0.00016; UVA + UVB: F(3, 18) = 1.336, p = 0.291. Asterisks denote statistically significant pairwise differences, based on *post-hoc* t-tests with unequal variances and Bonferroni correction (6 comparisons, p_adjusted ≈ 0.0083): *p_adjusted < 0.0083: **p_adjusted < 0.001. Absence of an asterisk indicates no significant difference between compared groups.

*A. thaliana* seedlings were grown under controlled conditions for a period of two weeks. Subsequently, the plants were exposed to UV in a specialized cabinet equipped with three distinct UV lamps. The plants were irradiated individually with UVA, UVB or UVC for various durations: 10, 30, or 120 minutes, respectively. The irradiance used was 12 W·m^-2^ and therefore the UV doses were of 7.2, 21.6 and 86.4 KJ·m^-2^ for 10, 30 and 120 min, respectively. A control group of non-irradiated plants was utilized to establish a baseline for the experiment. After the preparation of the corresponding acid extracts, the extracts were mixed with PMA. The PMA^red^ intensity color obtained was recorded for each sample 30 min after the initiation of the reduction reaction. As illustrated in **Figure 5**, the maximum absorption recorded for PMA^red^ was determined by averaging the values obtained from the various extracts for each type of UV radiation and at each designated time point, as compared to the values obtained for the control samples (0 min). For a comprehensive overview of the values collected for each extract at each radiation and condition, refer to the **Supplementary Material** (**Supplementary Figures S11**-**S15**).

The results demonstrated that for UVA, the redox activity of the extracts increased during the initial 10 min of exposure in comparison to that observed for the control plants that remained unexposed to UV (**Figure 5A**). For exposure times of 30 and 120 min, the reduction capacity drops, but still remains higher than the control. Overall ANOVA analysis of these values indicated differences between values (F(3,18) = 4.726, p = 0.013), but Bonferroni adjusted *post-hoc* tests failed to identify specific differences between pairs.

For UVC, the trend found was similar to that of UVA, with the redox activity increasing at the shortest exposure time and then decreasing with longer exposures (**Figure 5C**). As expected, the effect of UVC was more pronounced, as the decrease in absorption recorded for PMA^red^ was considerably greater for the extended exposure times, falling well below that observed for the control plants. In fact, the statistical analysis determined significant differences for the data at 30 and 120 min in relation to both the control and the value at 10 min.

Interestingly, the effect observed with UVB was the opposite of what had been seen thus far (**Figure 5B**). The redox activity decreased slightly for the first 10 min of radiation with respect to the control but increased for longer exposure times. In this case, the data at 120 min showed statistically significant differences compared to the other exposure times and, of course, compared to the control.

Finally, for the case of UVA+UVB, the data found seem to be maintained over time, since, although at 10 min the PMA^red^ absorbance signal increases, it decreases to control levels at 30 min and increases again at 120 min, although the increase in this case is not as considerable as that recorded for UVB alone (**Figure 5D**). This treatment was the only case in which the overall ANOVA analysis identified no significant differences.

## 4 Discussion

### 4.1 Suitability of PMA as an electrochromic sensor for redox activity in acidic plant extracts

The experimental findings presented above provide strong evidence that PMA can serve as an effective electrochromic sensor for assessing redox activity in acidic plant extracts. Specifically, both GSH and AsA, the two major non-enzymatic antioxidants involved in the plant defense system against oxidative stress, were shown to reduce PMA, producing a characteristic blue color. The linear increase in absorbance at 852 nm with increasing concentrations of either molecule supports the sensitivity and reliability of this detection system.

Importantly, under the experimental conditions established (PMA 10 mM in 10 mM HCl), both GSH and AsA exhibited indistinguishable UV-visible spectral profiles, with no interference from their respective oxidized forms (GSSG and DHA). This finding confirms that the electrochromic reaction is specific to the reduced forms of the antioxidants, which are biologically relevant in the context of redox homeostasis. Moreover, H_2_O_2_, one of the most studied reactive oxygen species in plants, did not induce any detectable reduction of PMA at the tested concentrations, reinforcing the selectivity of the method toward biological reductants rather than general oxidants.

Furthermore, when the minor antioxidants FAD, CA, and PH were evaluated, only CA exhibited a measurable reducing capacity toward PMA, displaying a response similar to that observed for AsA. However, it is important to note that this effect was only detectable at concentrations of 10 µM and above. According to the metabolomic data reported by Jaini et al. (2017), the content of caffeic acid and other catecholic compounds in *A. thaliana* wild-type plants under non-stress conditions does not reach micromolar levels. Indeed, it is approximately 600-fold lower than the concentrations tested here (Jaini et al., 2017). Consequently, within physiological contexts, the impact of catechols on PMA reduction can be regarded as negligible. In addition, no detectable reduction of PMA was observed in the presence of either FAD or phenol under identical conditions. This finding suggests that these classes of minor phenolic and flavinic compounds are improbable to interfere with PMA-based redox measurements in acidic plant extracts.

The observations presented here demonstrate that the PMA response in plant acid extracts is predominantly influenced by the total redox activity driven by the prevalent non-enzymatic antioxidants, GSH and AsA. The role of secondary metabolites such as flavins, phenolics, and catechols is not anticipated to be substantial, unless they attain unusually high concentrations under specific stress conditions.

This lack of specificity between GSH and AsA, although limiting in terms of individual quantification, is in fact advantageous for evaluating the overall redox potential of a sample. In complex biological matrices such as acid plant extracts, where both GSH and AsA coexist and contribute to the cellular redox activity, a combined readout provides a more integrative measure of the redox state. As such, the observed increase in PMA^red^ absorbance in response to abiotic stress can be interpreted as an elevation in redox activity, reflecting rapid changes in the AsA and GSH pools, consistent with the activation of antioxidant responses reported in the literature (Gupta et al., 2013, 2017; Pandey et al., 2017; Hasanuzzaman et al., 2020; Peco et al., 2020).

Taken together, these results validate the use of PMA as a simple, robust, and cost-effective electrochromic probe for monitoring dynamic changes in the redox status of plants, particularly under stress conditions. The proposed PMA-based assay signifies a substantial practical advancement in the high-throughput analysis of the plant redox state. In comparison with conventional methods for the quantification of AsA and GSH, the proposed approach exhibits several distinct advantages, namely: i) cost-effectiveness, as it does not necessitate the use of costly instrumentation or specific commercial enzymatic kits; ii) the capacity to analyze a greater number of samples in a more efficient manner, with a reaction time of merely 30 min, and compatibility with 96-well plate formats for the rapid and simultaneous analysis of a high volume of samples and iii) the absence of any expertise requirement, due to the simplicity of the test. Additionally, the method’s compatibility with acidic extraction procedures and its ability to detect biologically relevant antioxidant responses without interference from common ROS molecules position it as a valuable tool for screening physiological versus stress-induced redox changes in plant tissues.

### 4.2 PMA-based redox profiling reveals differential antioxidant responses to abiotic stress in *Arabidopsis thaliana*

Abiotic stresses such as salinity and UV are known to trigger complex physiological and biochemical responses in plants, including redox adjustments involving key antioxidants like GSH and ascorbic acid AsA. In this study, we evaluated the utility of a PMA-based electrochromic assay to assess the redox activity in acid extracts of *Arabidopsis thaliana* subjected to salinity and UV stress. Despite the simplicity of the assay, the results successfully revealed biologically meaningful trends between eustress and distress phases, aligning with previous knowledge on antioxidant dynamics in *A. thaliana* under moderate and severe stress conditions.

#### 4.2.1 Redox responses to salinity stress

Although our redox activity data for salinity did not reach statistical significance (Games-Howell, p > 0.05), the visual trend of increase and subsequent decrease is consistent with the literature describing biphasic stress (eustress/distress). For example, prolonged salt treatments (≥ 72) at concentrations ranging from 50 to 150 mM induce a significant accumulation of GSH and AsA in *A. thaliana*, along with enhanced activity of associated antioxidant enzymes (Foyer and Noctor, 2011; Horváth et al., 2020). Notably, the highest redox activity was observed at 150 mM NaCl, a concentration previously associated with peak antioxidant enzyme induction and maximal redox activity in *A. thaliana* (Horváth et al., 2020). Such responses are interpreted as part of a eustress adaptation, enabling plants to suffer oxidative damage while maintaining metabolic activity and growth.

Conversely, the decline in the redox activity found at 200 mM NaCl indicates a loss of efficiency in the plant’s ability to maintain its GSH and/or AsA reserves (distress). This is in agreement with reports describing a collapse in the antioxidant defense system at high salt concentrations, characterized by the depletion of GSH and AsA pools and accumulation of the oxidized forms (GSSG and DHA) (Gill and Tuteja, 2010; Horváth et al., 2020).

When considering other plant species, varying degrees of salt tolerance result in distinct redox responses (Ashraf and Harris, 2004; Flowers and Colmer, 2008; Munns and Tester, 2008). Salt-tolerant genotypes, such as the Jerba accession of *Cakile maritima* (Amor et al., 2006, 2007) and the Puget pea cultivar (Hernández et al., 1999; Hernández et al., 2001; Gómez et al., 2004), maintain or gradually increase GSH and AsA levels and efficiently recycle oxidized forms. In contrast, salt-sensitive genotypes like the Tabarka accession of *Cakile maritima* (Amor et al., 2006, 2007) or the Lincoln pea (Hernández et al., 1999, 2001; Gómez et al., 2004) show greater antioxidant depletion and oxidative imbalance. This reinforces the concept of an optimal window of stress intensity in *A. thaliana* where the antioxidant machinery is fully activated, but not yet overwhelmed. Beyond this threshold, as seen with 200 mM NaCl, the redox system collapses, transitioning from eustress to distress.

#### 4.2.2 Redox modulation under UV radiation

To explore a distinct abiotic stress involving direct redox signaling, we investigated UV radiation. UV spans 100–400 nm and includes UVA (315–400 nm), UVB (280–315 nm), and UVC (100–280 nm). In nature, plants are exposed to all UVA and approximately 100 times less UVB, as the ozone layer blocks most UVB and all UVC. The average daily radiation ranges established are from 900 to 1725 KJ·m^-2^ for UVA and from 2 to 12 KJ·m^-2^ for UVB (Kataria et al., 2014; Escobedo-Bretado et al., 2017). However, the dose varies according to season, altitude, and latitude, which is why in certain regions, such as southern Europe, peak doses of over 50 KJ·m^-2^ are reached (Aguilera et al., 2004; Martínez-Lozano et al., 2012). Despite UVA’s abundance, its damaging effects on plants are less studied compared to UVB. This is due to the abundance of information regarding the effects of UVB on cell damage and regulatory response (Verdaguer et al., 2017). Specifically, UV has been demonstrated to influence crop growth and yields (Sharma et al., 2019). Low-dose UVB has been observed to stimulate growth, while high-dose UVB radiation has been shown to retard it (Meyer et al., 2021). In contrast, the phenomenon under scrutiny has been demonstrated to exert an influence on a variety of physiological processes, including but not limited to photosynthesis, plant morphogenesis, and the synthesis of certain secondary metabolites (Rozema et al., 1997; Kataria et al., 2014; Shi and Liu, 2021; Sánchez-Correa et al., 2023). It has also been shown that UVB induces DNA damage and elevates the levels of ROS, such as H_2_O_2_ (Piri et al., 2011; Shi and Liu, 2021; Sánchez-Correa et al., 2023). As with other forms of abiotic stress, the plant activates the AOS mechanism to destroy the ROS generated by UVB. This results in an increase in the enzymes and molecules involved. Increases in AsA and GSH levels due to UVB have been documented for various plant species, including lettuce, cucumber (Liu et al., 2019, 2023) and *A. thaliana* (Gao and Zhang, 2008; Wang et al., 2010; Haskirli et al., 2021). UVC has been observed to induce detrimental effects on all forms of life (Sagan, 1973; Diffey, 1991). The underlying mechanism of this phenomenon has been investigated in *A. thaliana* (Danon and Gallois, 1998; Danon et al., 2004; Gao et al., 2008).

In our study, the PMA-based assay was sensitive to distinct responses depending on the radiation type and duration. UVA and UVC treatments exhibited a biphasic pattern: an early increase in redox activity followed by a decline with prolonged exposure, consistent with early activation and later exhaustion of antioxidant responses. The case of UVC was particularly striking, as extended exposure significantly suppressed redox activity below control levels, indicative of oxidative damage and cellular distress. These findings are in agreement with prior studies showing that UVC imposes severe genotoxic and oxidative stress in *A. thaliana*, leading to rapid ROS accumulation and cellular dysfunction (Danon and Gallois, 1998; Danon et al., 2004; Gao and Zhang, 2008).

UVB elicited a unique response. Redox activity decreased slightly after 10 min exposure but increased significantly at 30 and 120 min, with the latter showing the highest PMA^red^ signal among all treatments. In order to enhance comprehension of the behavior exhibited by *A. thaliana* in response to UVB, it is imperative to acknowledge the pivotal role of the time at which the dose is administered in the analysis of the plant’s response. It has been demonstrated that low doses administered over a short period of time, or a high dose accumulated over a long period of time, permit the plant to acclimatize (Jenkins, 2009; Hideg et al., 2013; Mmbando, 2023; Gastélum-Estrada et al., 2024). In these cases, the response consists mainly of the expression of genes responsible for the synthesis of metabolites that enhance the plant’s radiation tolerance (Bechtold et al., 2008; Shi and Liu, 2021; Jansen et al., 2022). This is exemplified by the UV resistance receptor locus 8 (UVR8), which plays a role in the promotion of flavonoid biosynthesis, molecules that possess the capacity to absorb UV (Jenkins, 2009; Shi and Liu, 2021; Jansen et al., 2022). However, when a high dose is applied over a short period of time, the plant does not have time to acclimatize, and what occurs is an emergency antioxidant response (Kataria et al., 2014; Haskirli et al., 2021) where the mechanism that is activated is not the expression of genes such as UVR8, but rather the activation of specific antioxidant pathways that cause the enhancement of the levels of antioxidant molecules within plants (Shiu and Lee, 2005; Hideg et al., 2013). Considering these findings, UVB particularly at low doses, is regarded as relatively innocuous and not an antioxidant generator for *A. thaliana* (Hectors et al., 2007; Meyer et al., 2021), inducing eustress conditions rather than distress. On the contrary, Haskirli et al. (2021), determined that, after 180 min (with a dose of 92 KJ·m^-2^) of exposure to UVB the GSH content increases by 81% with respect to the initial content (Haskirli et al., 2021). Accordingly, the findings on our study indicate that the effect observed in the redox activity of *A. thaliana* after exposure to UVB at 12 W·m^-2^ in short times is consistent with the activation of the emergency antioxidant machinery with the corresponding increase of AsA and GSH concentrations, thereby explaining the high absorbances recorded for PMA^red^.

Finally, the combined UVA+UVB treatment yielded a non-monotonic pattern: a moderate increase at 10 min, a drop to control levels at 30 min, and a recovery at 120 min, although not as marked as with UVB alone. The absence of significant differences may indicate antagonistic or buffering interactions between UVA and UVB effects, consistent with the complexity of plant responses to full-spectrum UV radiation.

## 5 Conclusion

In summary, the findings of this study underscore the promise of PMA as a probe to establish a correlation between plant redox activity and abiotic stressors. Under salinity, the redox activity of moderate concentrations resulted in an increase followed by a decrease at the highest levels. Despite this trend, the results were non-significant and not statistically conclusive. However, this highlights the potential of the PMA assay for monitoring subtle shifts in the redox state. The validity of the method was further demonstrated by the statistically significant changes observed under UV radiation. It is noteworthy that UVC has been demonstrated to be associated with a significant decrease in redox activity, which is indicative of its lethal effect. Conversely, UVA has been observed to maintain redox activity, indicating the potential for an adaptive eustress response. The most significant aspect of our research is the observation of a remarkable increase in the redox activity of *A. thaliana* under UVB, indicating the activation of an emergency antioxidant response due to the accumulation of high irradiance over a short period.

This novel approach signifies a substantial simplification in the evaluation of plant metabolic capacity to abiotic stresses and unveils novel avenues for the study of stress responses in plants.

## Data Availability

The datasets presented in this study can be found in online repositories. The names of the repository/repositories and accession number(s) can be found below: https://doi.org/10.5281/zenodo.17795112.
