# Supplementary material for: Electrochromic polyoxometalates for sensing abiotic stress in plants
Source: Front Plant Sci. 2026 Jan 2;16:1672784. doi: 10.3389/fpls.2025.1672784 (PMC12807894; doi:10.3389/fpls.2025.1672784)
Supplement: Supplementary file 1 [file DataSheet1.docx]

Supplementary Material

**
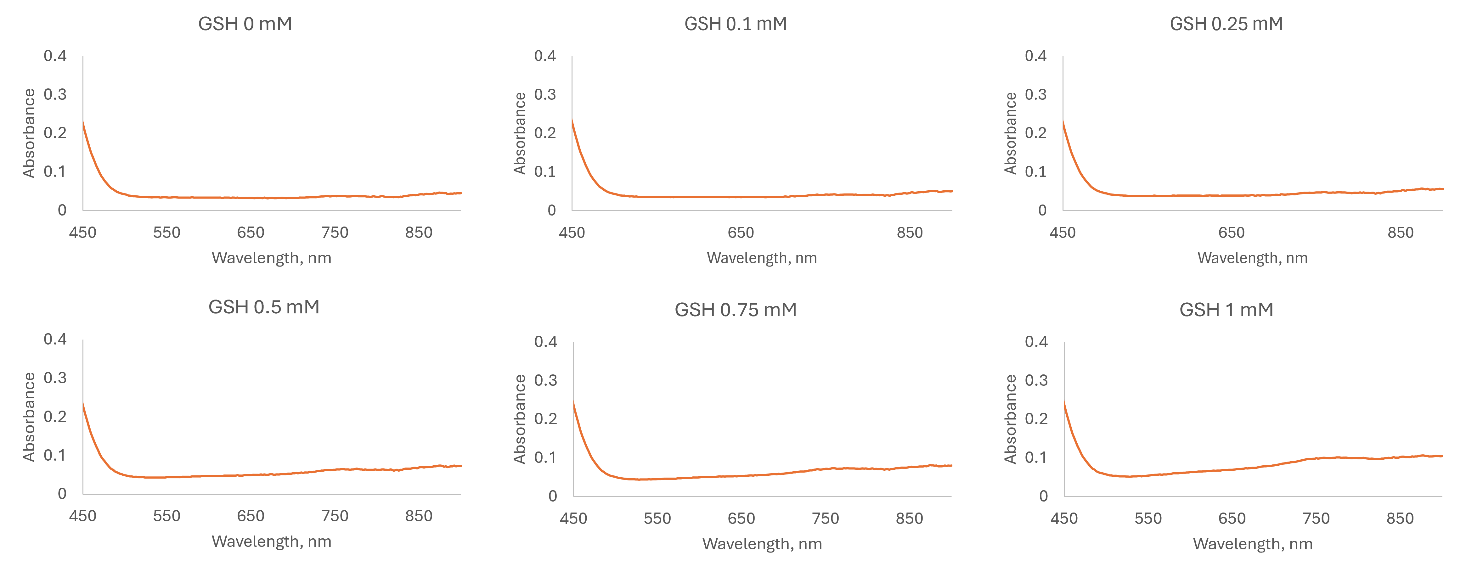
**

**Supplementary Figure 1.** UV-visible spectra of varying GSH concentrations with 1 mM synthetic POM (PMO). Spectra were recorded from 450 to 900 nm after 30 minutes of adding 1 mM PMO to different concentrations of reduced glutathione (GSH). Bands are centered at 780 nm. Each curve represents the average of 3 technical replicates measurements.


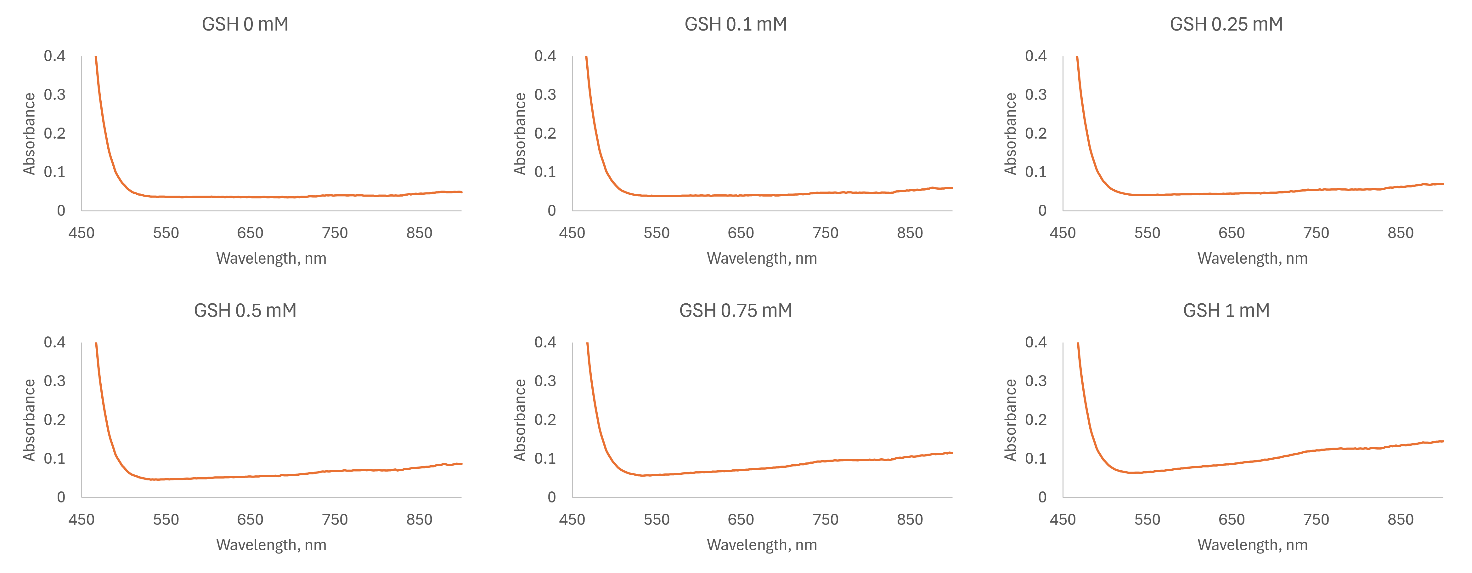


**Supplementary Figure 2.** UV-visible spectra of varying GSH concentrations with 5 mM synthetic POM (PMO). Spectra were recorded from 450 to 900 nm after 30 minutes of adding 5 mM PMO to different concentrations of reduced glutathione (GSH). Bands are centered at 780 nm. Each curve represents the average of 3 technical replicates measurements.

**
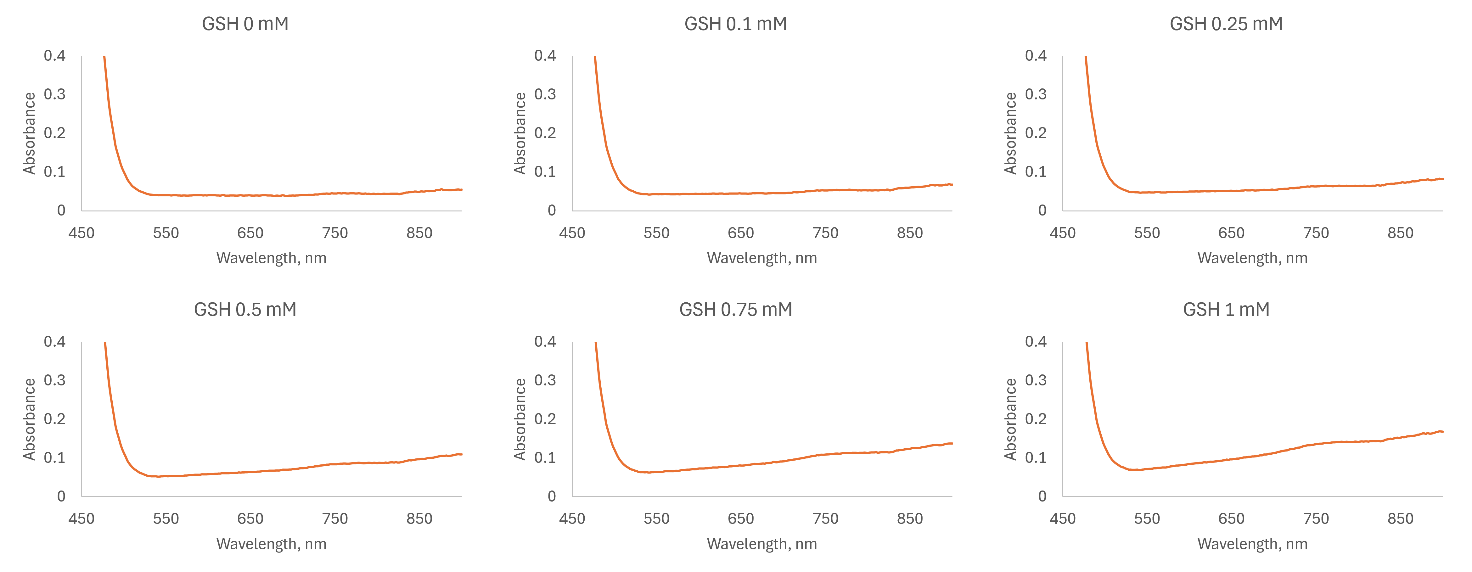
**

**Supplementary Figure 3.** UV-visible spectra of varying GSH concentrations with 10 mM synthetic POM (PMO). Spectra were recorded from 450 to 900 nm after 30 minutes of adding 10 mM PMO to different concentrations of reduced glutathione (GSH). Bands are centered at 780 nm. Each curve represents the average of 3 technical replicates measurements.


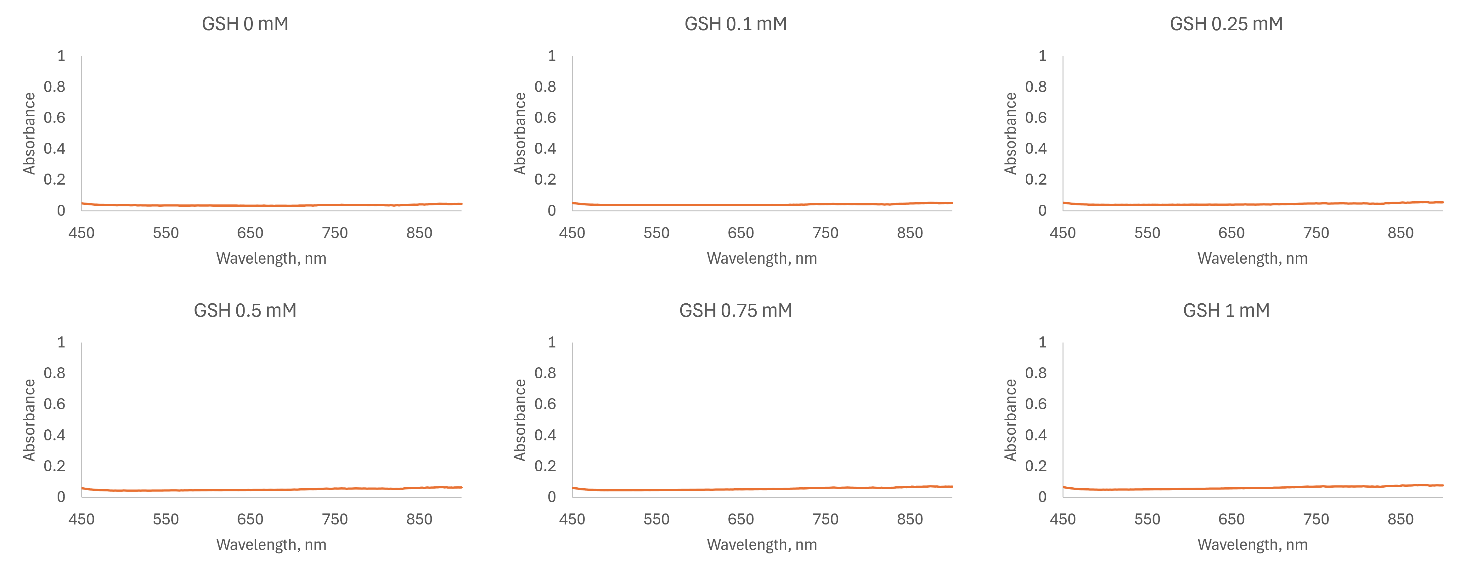


**Supplementary Figure 4.** UV-visible spectra of varying GSH concentrations with 1 mM phosphomolibdic acid hydrate (PMA). Spectra were recorded from 450 to 900 nm after 30 minutes of adding 1 mM PMA to different concentrations of reduced glutathione (GSH). Bands are centered at 852 nm. Each curve represents the average of 3 technical replicates measurements.


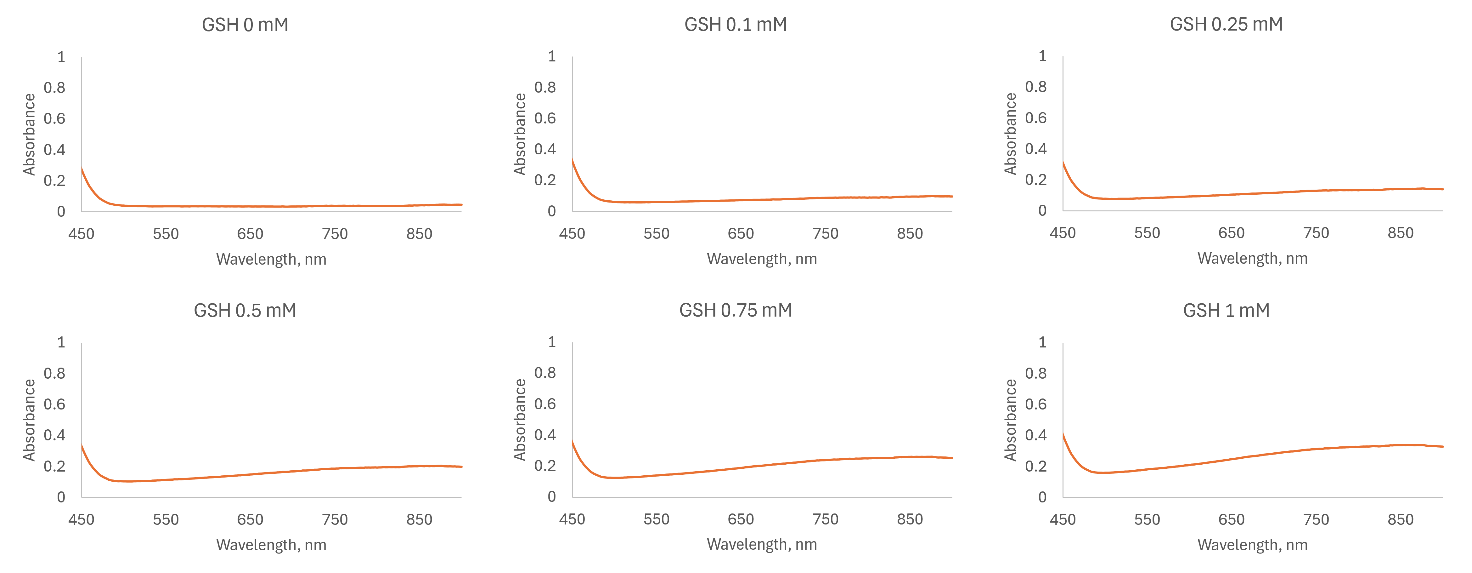


**Supplementary Figure 5.** UV-visible spectra of varying GSH concentrations with 5 mM phosphomolibdic acid hydrate (PMA). Spectra were recorded from 450 to 900 nm after 30 minutes of adding 5 mM PMA to different concentrations of reduced glutathione (GSH). Bands are centered at 852 nm. Each curve represents the average of 3 technical replicates measurements.


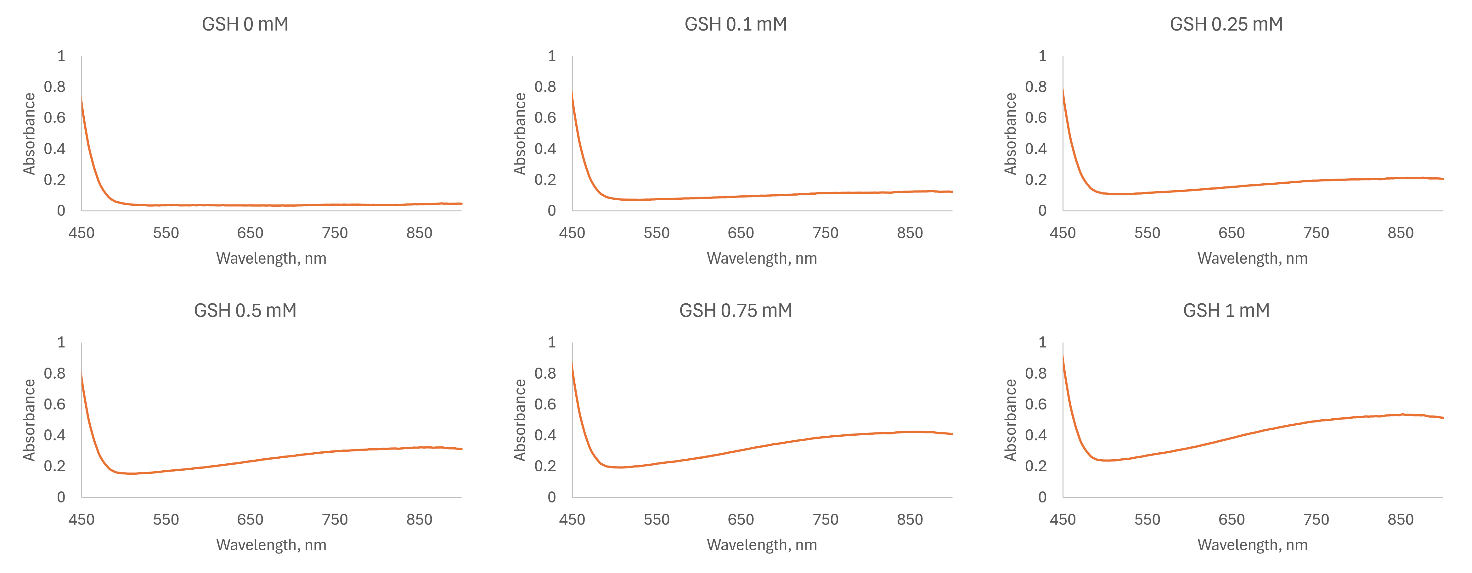


**Supplementary Figure 6.** UV-visible spectra of varying GSH concentrations with 10 mM phosphomolibdic acid hydrate (PMA). Spectra were recorded from 450 to 900 nm after 30 minutes of adding 10 mM PMA to different concentrations of reduced glutathione (GSH). Bands are centered at 852 nm. Each curve represents the average of 3 technical replicates measurements.


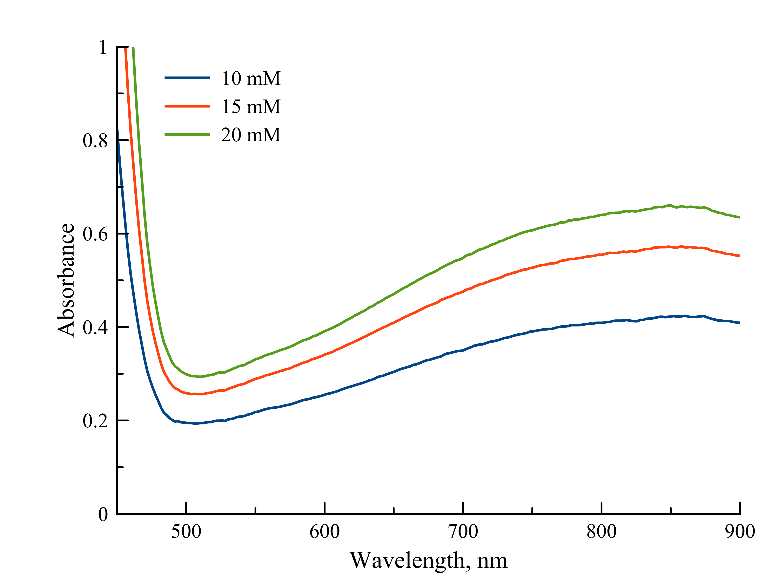


**Supplementary Figure 7.** UV-visible spectra profiles for 1 mM reduced glutathione (GSH) samples with different phosphomolybdic acid hydrate (PMA) concentrations. Spectra recorded from 450 to 900 nm show the profile for 1 mM GSH samples in the presence of 10 mM PMA (blue line), 15 mM PMA (orange line), and 20 mM PMA (green line). Each curve represents the average of 3 technical replicates measurements.


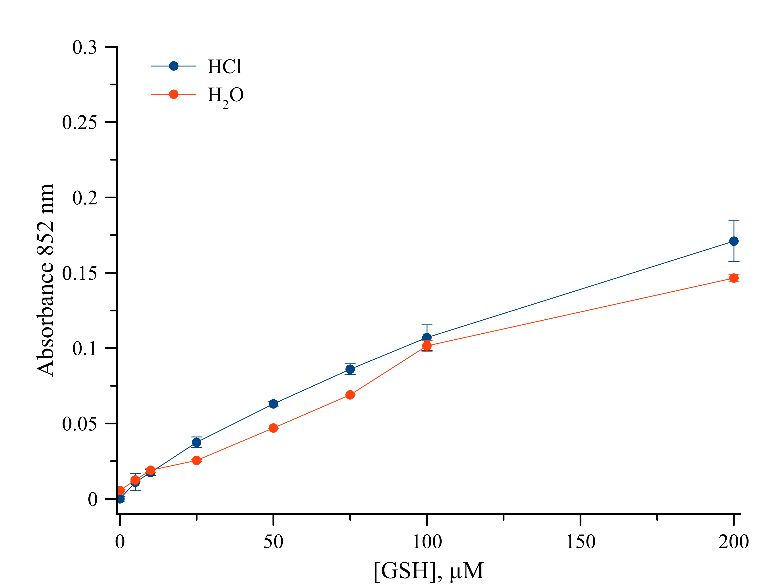


**Supplementary Figure 8.** Comparison of maximum absorbance at 852 nm for PMA in water and HCl across various GSH concentrations. The maximum absorbance at 852 nm was registered for PMA in H_2_O (red data points) and HCl (blue data points) at each reduced glutathione (GSH) concentration ranging from 5 to 200 µM. Each data point represents the mean of 3 technical replicates. Error bars represent standard deviations.


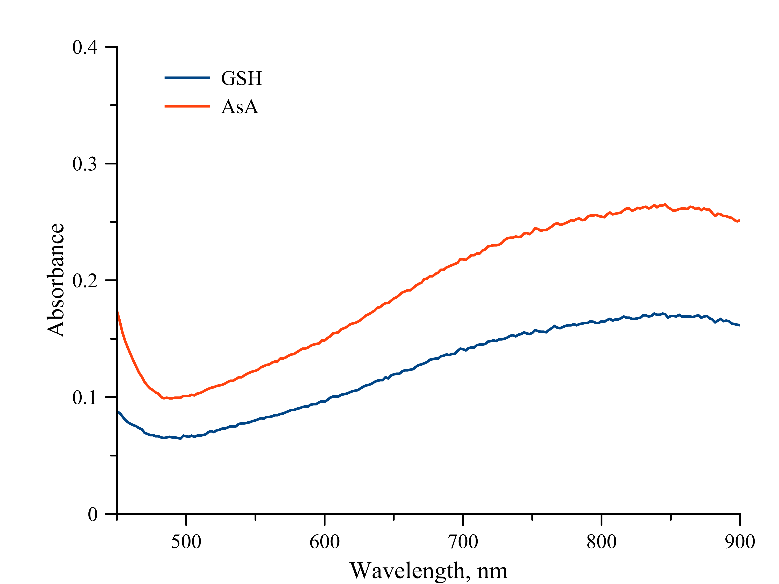


**Supplementary Figure 9.** UV-visible spectra profile comparison for 10 mM PMA with 200 µM GSH and 200 µM Ascorbic Acid (AsA). Spectra recorded from 450 to 900 nm show the profile for 10 mM PMA in the presence of 200 µM reduced glutathione (GSH, red line) and 200 µM ascorbic acid (AsA, blue line). Each curve represents the average of 3 technical replicates measurements.

**
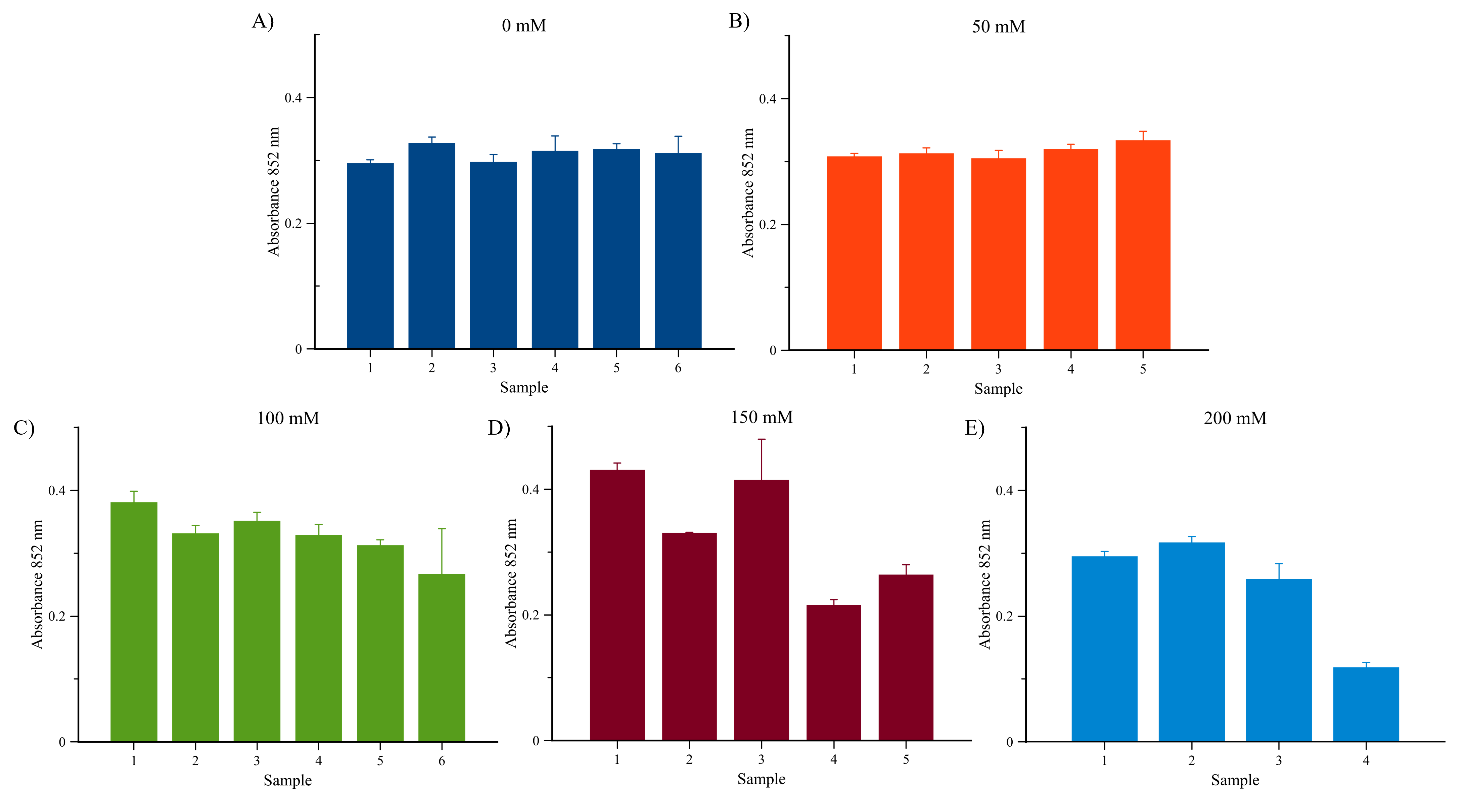
**

**Supplementary Figure 10.** Maximum absorbance at 852 nm from individual *Arabidopsis thaliana* extracts exposed to varying NaCl concentrations. Maximum absorbance at 852 nm was collected from acidic plant extracts after 5 days of exposure to 0 (A), 50 (B), 100 (C), 150 (D), and 200 (E) mM NaCl concentrations. Each bar represents the mean absorbance of a single biological extract, calculated from its technical triplicates. The number of biological extracts (n) for each treatment was: 6 for 0 and 100 mM NaCl; 5 for 50 and 150 mM NaCl; and 4 for 200 mM NaCl. Error bars represent the standard deviation of the technical triplicates for each individual extract.

**
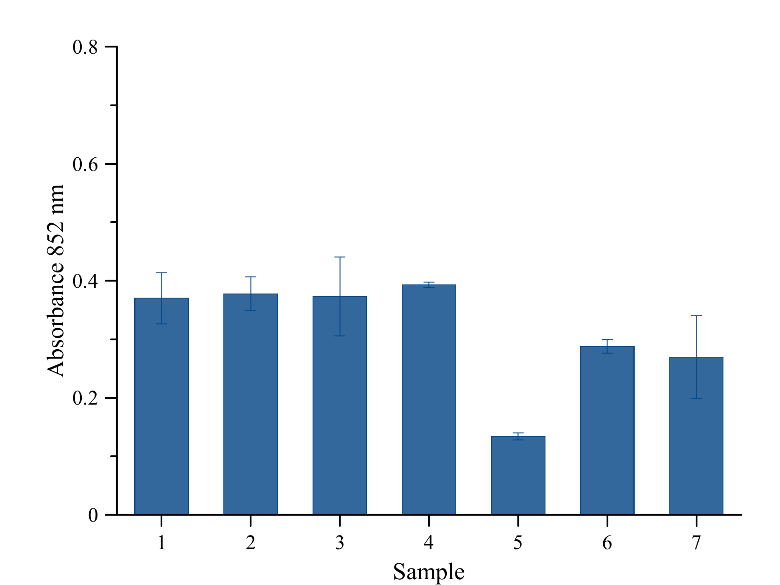
**

**Supplementary Figure 11.** Maximum absorbance at 852 nm from individual control *Arabidopsis thaliana* extracts (0 min UV). Maximum absorbance at 852 nm was collected from acidic extracts of *A. thaliana* plants not exposed to UV radiation. Each bar represents the mean absorbance of a single biological extract, calculated from its technical triplicates. A total of 7 biological extracts (n=7) were analyzed for this control group. Error bars represent the standard deviation of the technical triplicates for each individual extract.

*
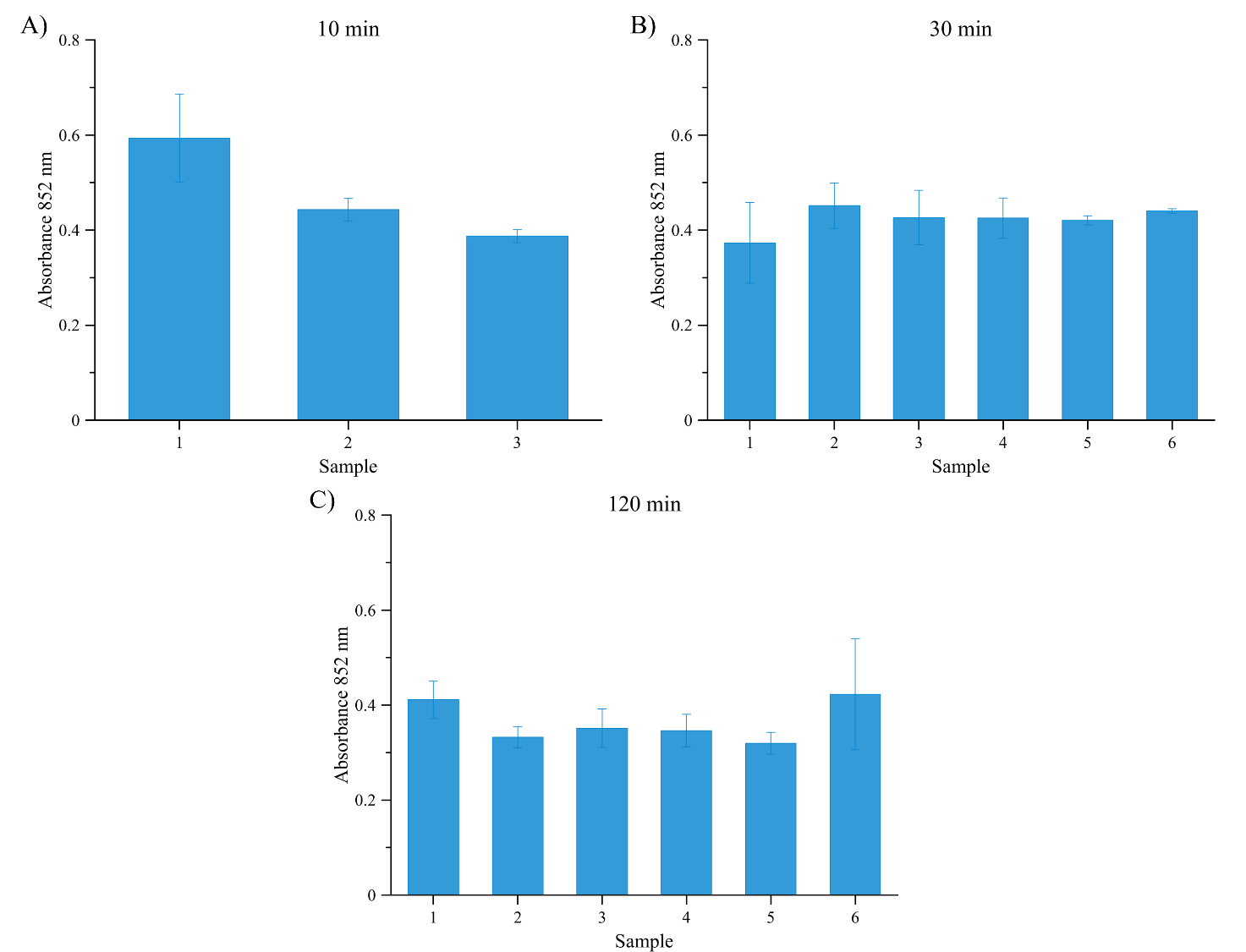
*

**Supplementary Figure 12.** Maximum absorbance at 852 nm from individual *Arabidopsis thaliana* extracts treated with UVA radiation. Maximum absorbance at 852 nm was collected from acidic extracts of *A. thaliana* plants after exposure to UVA for 10 (A), 30 (B), and 120 (C) minutes. Each bar represents the mean absorbance of a single biological extract, calculated from its technical triplicates. The number of biological extracts (n) for each treatment was: 3 for 10 min, 6 for 30 & 120 min. Error bars represent the standard deviation of the technical triplicates for each individual extract.


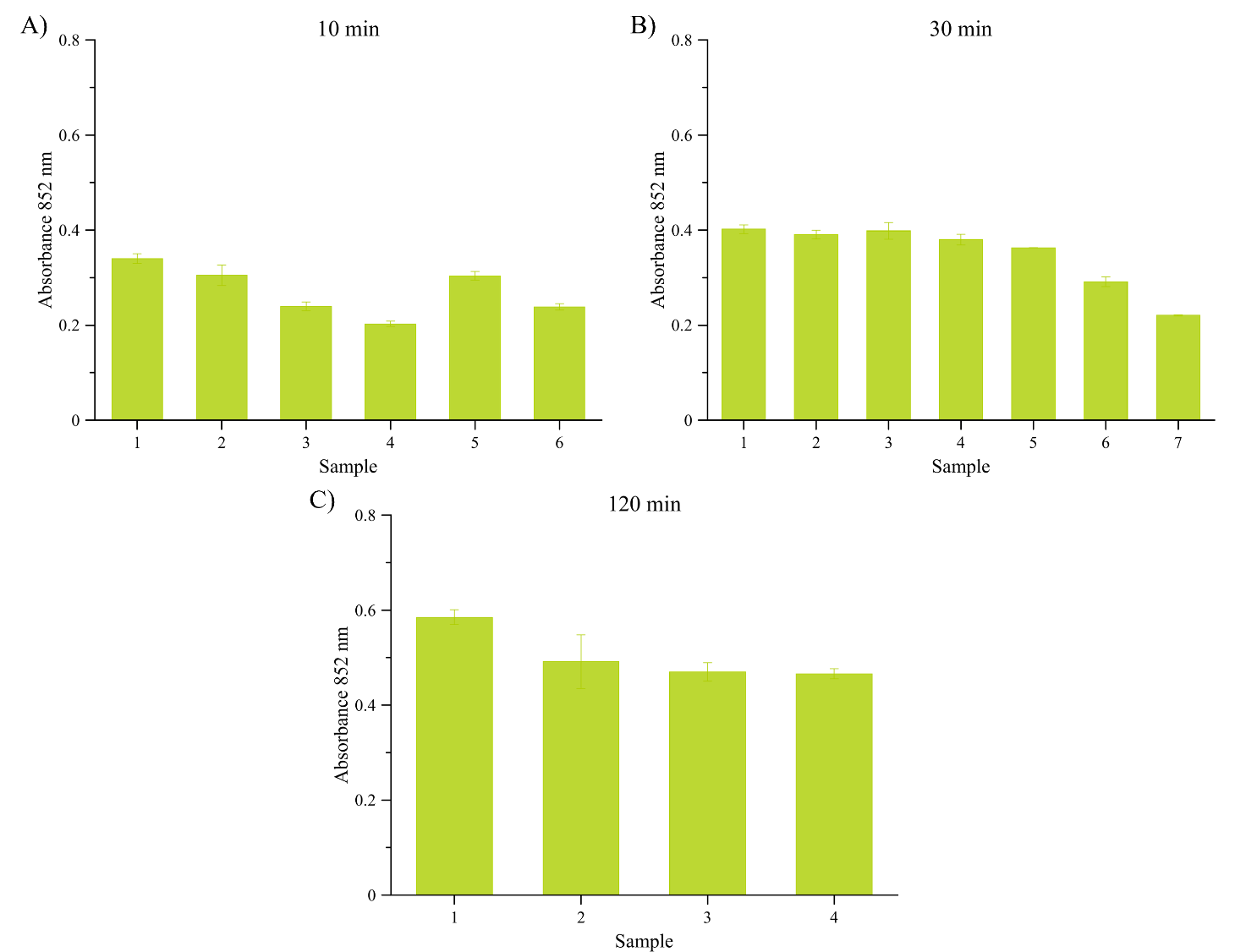


**Supplementary Figure 13.** Maximum absorbance at 852 nm from individual *Arabidopsis thaliana* extracts treated with UVA radiation. Maximum absorbance at 852 nm was collected from acidic extracts of *A. thaliana* plants after exposure to UVB for 10 (A), 30 (B), and 120 (C) minutes. Each bar represents the mean absorbance of a single biological extract, calculated from its technical triplicates. The number of biological extracts (n) for each treatment was: 6 for 10 min, 7 for 30 min, 4 for 120 min. Error bars represent the standard deviation of the technical triplicates for each individual extract.


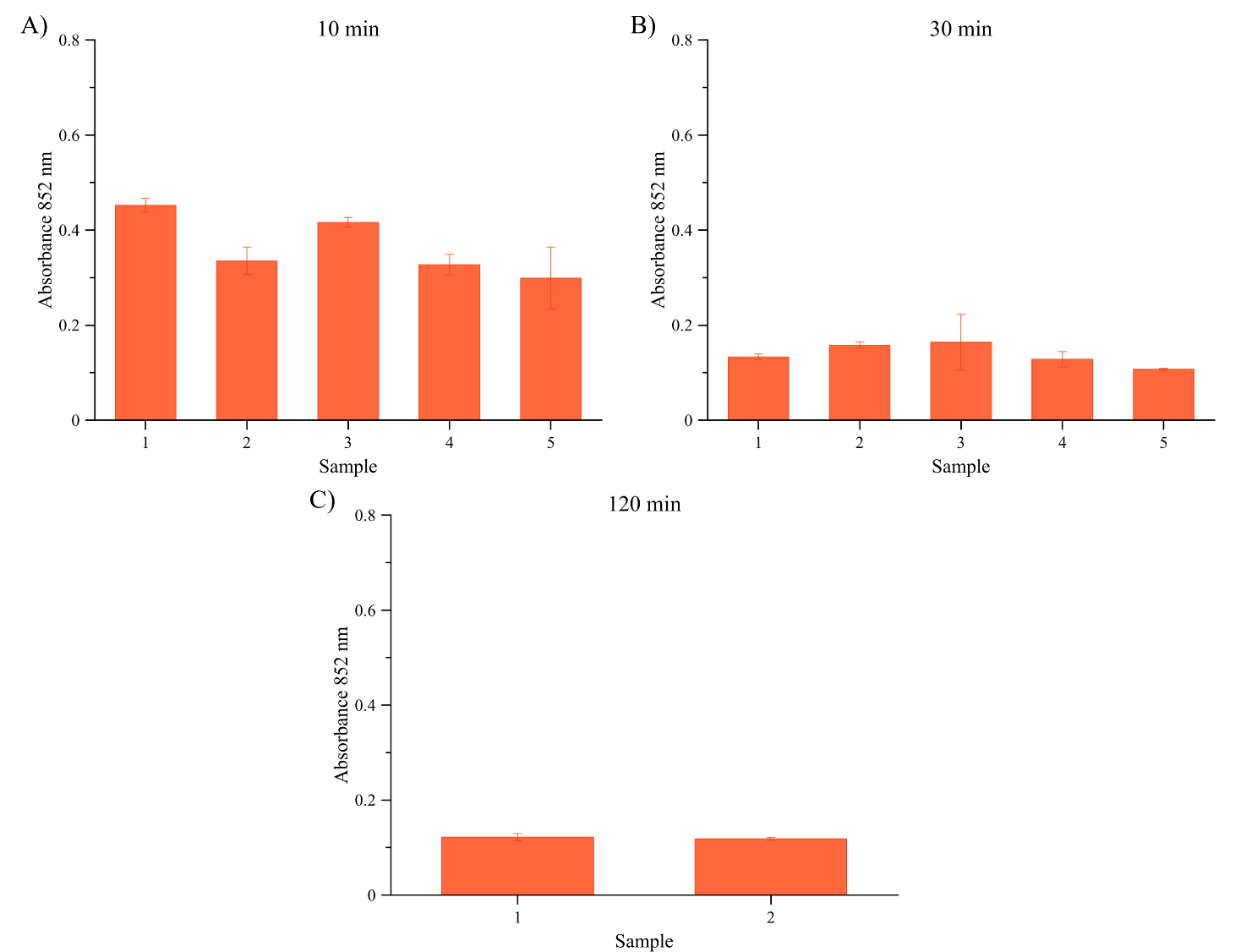


**Supplementary Figure 14.** Maximum absorbance at 852 nm from individual *Arabidopsis thaliana* extracts treated with UVA radiation. Maximum absorbance at 852 nm was collected from acidic extracts of *A. thaliana* plants after exposure to UVC for 10 (A), 30 (B), and 120 (C) minutes. Each bar represents the mean absorbance of a single biological extract, calculated from its technical triplicates. The number of biological extracts (n) for each treatment was: 5 for 10 min & 30 min, 2 for 120 min. Error bars represent the standard deviation of the technical triplicates for each individual extract.

**
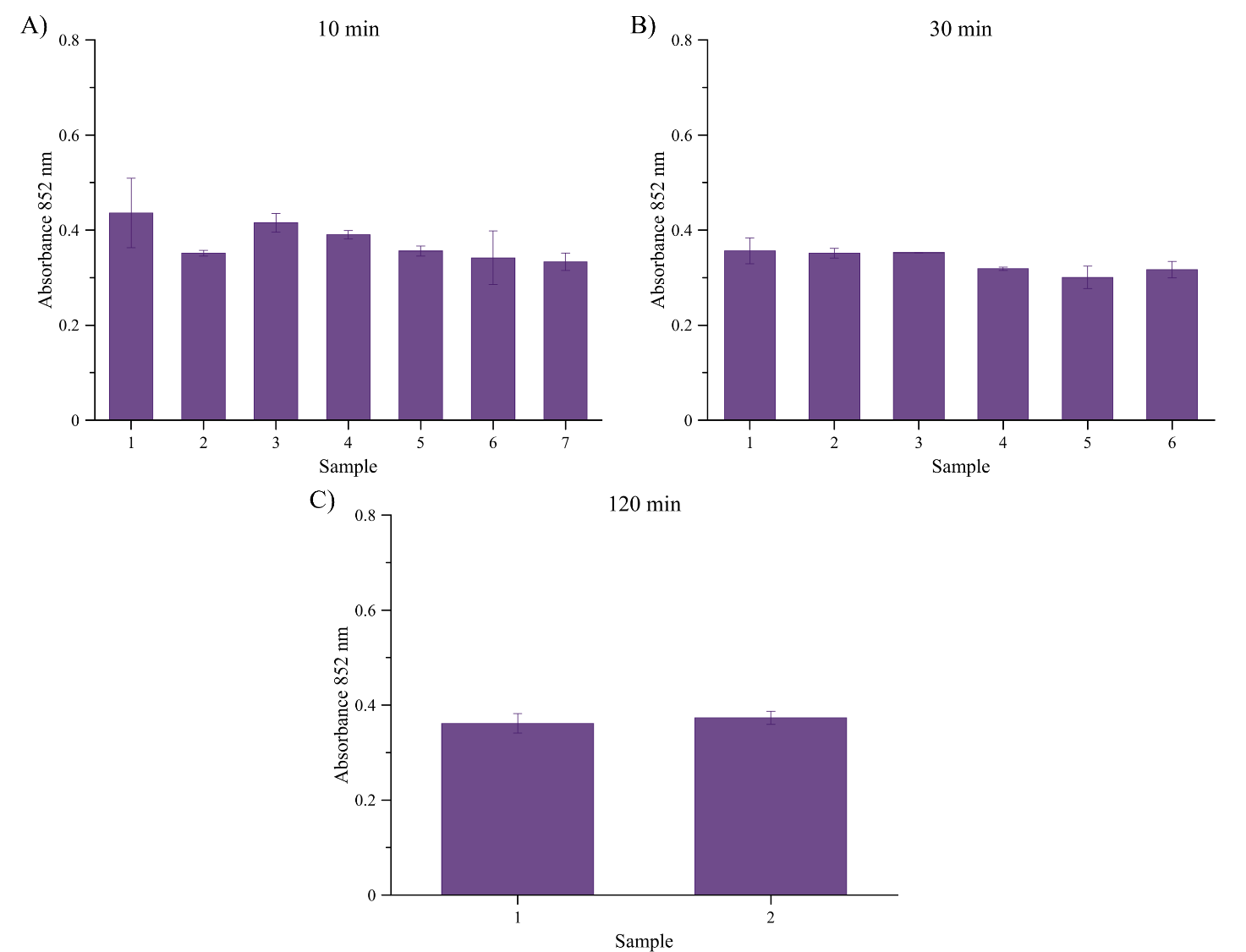
**

**Supplementary Figure 15.** Maximum absorbance at 852 nm from individual *Arabidopsis thaliana* extracts treated with UVA radiation. Maximum absorbance at 852 nm was collected from acidic extracts of *A. thaliana* plants after exposure to UVA+UVB for 10 (A), 30 (B), and 120 (C) minutes. Each bar represents the mean absorbance of a single biological extract, calculated from its technical triplicates. The number of biological extracts (n) for each treatment was: 7 for 10 min, 6 for 30 min, 2 for 120 min. Error bars represent the standard deviation of the technical triplicates for each individual extract.
